# Fabricating Polymer/Surfactant/Cyclodextrin Hybrid Particles for Possible Nose-to-Brain Delivery of Ropinirole Hydrochloride: In Vitro and Ex Vivo Evaluation

**DOI:** 10.3390/ijms25021162

**Published:** 2024-01-18

**Authors:** Elmina-Marina Saitani, Natassa Pippa, Diego Romano Perinelli, Aleksander Forys, Paraskevi Papakyriakopoulou, Nefeli Lagopati, Giulia Bonacucina, Barbara Trzebicka, Maria Gazouli, Stergios Pispas, Georgia Valsami

**Affiliations:** 1 Department of Pharmacy, School of Health Sciences, National and Kapodistrian University of Athens, Panepistimiopolis, 15771 Zografou, Greece; elminasait@pharm.uoa.gr (E.-M.S.); natpippa@pharm.uoa.gr (N.P.); ppapakyr@pharm.uoa.gr (P.P.); 2 School of Pharmacy, Chemistry Interdisciplinary Project (CHIP), University of Camerino, Via Madonna delle Carceri, 62032 Camerino, Italy; diego.perinelli@unicam.it (D.R.P.); giulia.bonacucina@unicam.it (G.B.); 3 Centre of Polymer and Carbon Materials, Polish Academy of Sciences, 34, M. Curie-Skłodowskiej St, 41-819 Zabrze, Poland; aforys@cmpw-pan.pl (A.F.); barbara.trzebicka@cmpw-pan.edu.pl (B.T.); 4 Laboratory of Biology, Department of Basic Medical Science, School of Medicine, National and Kapodistrian University of Athens, 11527 Athens, Greece; nlagopati@med.uoa.gr (N.L.); mgazouli@med.uoa.gr (M.G.); 5 Biomedical Research Foundation, Academy of Athens, 11527 Athens, Greece; 6 Theoretical and Physical Chemistry Institute, National Hellenic Research Foundation, 48 Vassileos Constantinou Avenue, 11635 Athens, Greece; pispas@eie.gr

**Keywords:** poloxamer 407, cyclodextrins, surfactant, ropinirole hydrochloride, nose-to-brain transport, Franz type diffusion cells, ex vivo nasal permeability, cryo-TEM images

## Abstract

Ropinirole is a non-ergolinic dopamine agonist used to manage Parkinson’s disease and it is characterized by poor oral bioavailability. This study aimed to design and develop advanced drug delivery systems composed of poloxamer 407, a non-ionic surfactant (Tween 80), and cyclodextrins (methyl-β-CD or hydroxy-propyl-β-CD) for possible brain targeting of ropinirole after nasal administration for the treatment of Parkinson’s disease. The hybrid systems were formed by the thin-film hydration method, followed by an extensive physicochemical and morphological characterization. The in vitro cytotoxicity of the systems on HEK293 cell lines was also tested. In vitro release and ex vivo mucosal permeation of ropinirole were assessed using Franz cells at 34 °C and with phosphate buffer solution at pH 5.6 in the donor compartment, simulating the conditions of the nasal cavity. The results indicated that the diffusion-controlled drug release exhibited a progressive increase throughout the experiment, while a proof-of-concept experiment on ex vivo permeation through rabbit nasal mucosa revealed a better performance of the prepared hybrid systems in comparison to ropinirole solution. The encouraging results in drug release and mucosal permeation indicate that these hybrid systems can serve as attractive platforms for effective and targeted nose-to-brain delivery of ropinirole with a possible application in Parkinson’s disease. Further ex vivo and in vivo studies to support the results of the present work are ongoing.

## 1. Introduction

Parkinson’s disease (PAD) is one of the most prevalent neurodegenerative disorders, affecting movement features, such as bradykinesia, rigidity, postural instability, and resting tremor, and non-motor features, including olfactory loss, sleep dysfunction, autonomic dysfunction, cognitive impairment, and psychiatric disturbances [1]. Patients with PAD gradually develop non-motor symptoms many years before the appearance of motor symptoms. The disorder’s prevalence augments with age [2], and its pathophysiology is characterized by the progressive destruction of dopaminergic nerve cells in the substantia nigra, an area located in the midbrain, which is of paramount importance for the regulation of movement [1]. The therapeutic regimen for PAD is based on the symptoms of each patient and it requires an evaluation of the risk–benefit ratio.

Up to this moment, most available drugs on the market for PAD are given by the oral, intravenous, or intramuscular routes of administration. However, hepatic first-pass metabolism, enzymatic degradation in the gastrointestinal (GI) tract, plasma protein binding, as well as the presence of the blood–brain barrier (BBB) and low brain permeability reduce the drug’s bioavailability and delay its delivery to the brain through the blood [3]. These obstacles confirm the necessity of an alternative route to overcome these limitations.

The sublingual and buccal mucosa were recently proposed as alternative routes of administration [4,5,6,7], presenting improved bioavailability at lower doses compared to oral formulations due to the avoidance of hepatic and GI first-pass metabolism. These routes are non-invasive, easy to administer, and provide a more rapid onset of action compared to the oral route. They are suitable for late-stage Parkinson’s disease patients with swallowing difficulties and gastric motility issues. Additionally, they are advantageous for drugs susceptible to pH and enzymatic degradation in the gastrointestinal tract [8,9].

To this end, the nasal route is most promising in terms of overcoming the drawbacks of limited brain accessibility of drug molecules during peripheral administration [3]. The highlighted advantages of the nose-to-brain transport route, such as the neuronal connection of the olfactory region with the central nervous system, circumventing the BBB, avoidance of first-pass metabolism, more precise drug targeting, safety, patient-friendly route of administration, acceleration of drug delivery process, reduced systemic side effects and non-intrusiveness, have proved that the intranasal route is a comfortable and suitable approach for the development of nasally administered formulations for PAD and a promising way to achieve therapeutic concentrations in the brain [10].

In recent years, researchers have explored the potential of the nasal route of administration as an alternative approach for managing Parkinson’s disease [11,12,13,14,15]. Lin et al. [13] fabricated a thermoresponsive gel containing 20% P407, 18% poloxamer 188, 1% polyethylene glycol 6000 and 3% hydroxypropyl-β-CD (HPβCD) for the intranasal administration of an antiparkinsonian drug, rhynchophylline. Imran et al. [12] developed a mucoadhesive microemulsion for the efficient delivery of silymarin, enhancing behavioral and biochemical parameters and reducing inflammatory markers in treated rats. Kim et al. [14] presented an innovative approach to enhance mitochondrial function and provide protection against Parkinson’s disease through the nasal route of administration. A mucoadhesive nasal thermosensitive gel, formulated by combining P407 with chitosan, was prepared by Sridhar et al. [15] to improve the bioavailability and brain concentration of selegiline hydrochloride, which is an anti-Parkinson’s agent.

Polymers have attracted the attention of scientists for several decades due to their ability to self-assemble into various nanostructures and morphologies, making them potential multifunctional candidates as carriers for active pharmaceutical ingredients (APIs), genes, and other bioactive compounds, either alone or in combination with other biocompatible molecules [16,17].

Pluronics, also referred as poloxamers, are amphiphilic triblock copolymers of the poly(ethylene oxide)-b-poly(propylene oxide)-b-poly(ethylene oxide) (PEO-PPO-PEO) type that have received approval for pharmaceutical application by the U.S. Food and Drug Administration [18]. Poloxamer 407 (P407) is an amphiphilic triblock copolymer consisting of a central hydrophobic chain of poly(propylene oxide) (PPO) of 1200 kDa molecular weight and 70% content of hydrophilic chains of poly(ethylene oxide) (PEO) [18].

Cyclodextrins (CDs) [19,20,21,22,23] and non-ionic surfactants [21,24] have been recognized in the literature for their potential as drug enhancers in nasal drug delivery. CDs have a truncated cone shape, with their lipophilic inner cavities surrounded by hydrophilic outer surfaces, allowing them to form inclusion complexes with a variety of molecules through non-covalent interactions [25,26]. In addition, polysorbate 80, also known as Tween 80 (Tw80), consists of polyoxyethylene sorbitan monooleate, which is a fatty acid ester of polyoxyethylene sorbitan. It is commonly used as an emulsifier, dispersant, and stabilizer, and is considered one of the most frequently used non-ionic surfactants in the industry [27].

Τhe beneficial effect arising from the combination of polymers with surfactants or polymers with CDs has been extensively reported in other studies. This effect is attributed to their encompassing the physicochemical and thermotropic properties of both classes of materials. More specifically, the complexation of polymers and CDs offers several advantages, including enhanced encapsulation efficiency and drug loading values than those achieved with pristine drugs [28,29,30,31], improved activity/efficacy [28,30,32,33], superior biocompatibility [32,34,35,36], greater release profile [20], long-term stability [30,36], improved inhibition efficacy against targeted cells [34], excellent rheological characteristics [34], and alleviated toxic effects [30,33,35]. Similarly, among the advantages that polymer–surfactant systems offer are enhanced bioavailability [37,38,39,40,41], improved drug targeting capability [38,40], ameliorated activity [39,40], long-term stability [40], and superior release profile [40]. Taking into account all the mentioned properties, it is worth examining the combination of biocompatible block copolymers with surfactants and CDs to design and develop complex structures that represent a new class of advanced nose-to-brain drug delivery systems.

To this end, ropinirole hydrochloride (RH) was selected as the model drug for the present study. It belongs to the category of non-ergolinic dopamine agonists, presenting affinity for both D2 and D3 receptors [42]. It is administered as monotherapy or as an adjunctive combination with levodopa, reducing levodopa’s dose [43]. Nevertheless, when RH is given orally, it presents poor bioavailability due to extensive first-pass hepatic metabolism, ranging from 34 to 57% [43]. Subsequently, alternative routes and drug delivery platforms are needed to ameliorate its efficacy in clinical practice.

**Figure 1:**
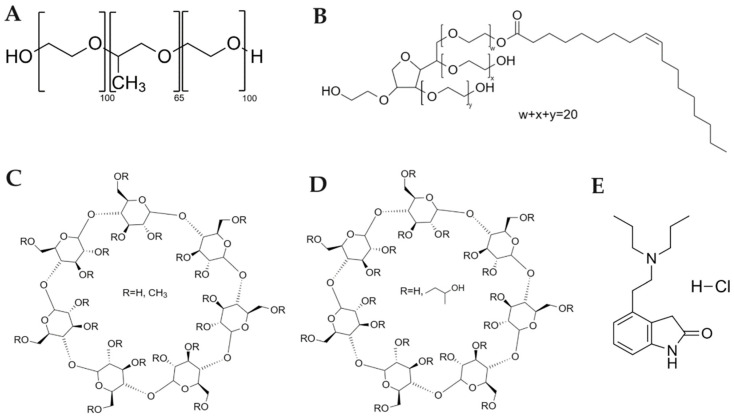
Chemical structures of (**A**) poloxamer 407, (**Β**) Tween 80, (**C**) methyl-β-cyclodextrin, (**D**) hydroxypropyl-β-cyclodextrin, and (**E**) ropinirole hydrochloride.

The chemical structures of the utilized components are presented Figure 1.

To the best of the authors’ knowledge, this is the first report presented in the literature of using hybrid nanoparticles composed of P407, a non-ionic surfactant (Tw80), and CDs (MβCD or HPβCD) for the development of nasal drug delivery systems and possible brain targeting of RH in the treatment of PAD. The prepared systems (in colloidal dispersion) were extensively characterized using several techniques, such as differential scanning calorimetry, dynamic light scattering, electrophoretic light scattering, microcalorimetry analysis, high-resolution ultrasound spectroscopy, fluorescence spectroscopy, surface tension measurements, and cryogenic transmission electron microscopy, to extract information about the morphological and physicochemical properties of the systems. Furthermore, in vitro cytotoxicity studies were conducted, while the release of RH from the developed systems was assessed in vitro using diffusion Franz cells and a regenerated cellulose membrane as a diffusion barrier under pH and temperature conditions simulating those of the nasal cavity. Proof-of-concept ex vivo experiments were also carried out to investigate the permeation profile of RH-containing systems in rabbit nasal mucosa.

## 2. Results and Discussion

### 2.1. Interactions between the Materials of Hybrid Systems in the Solid State

**Figure 2:**
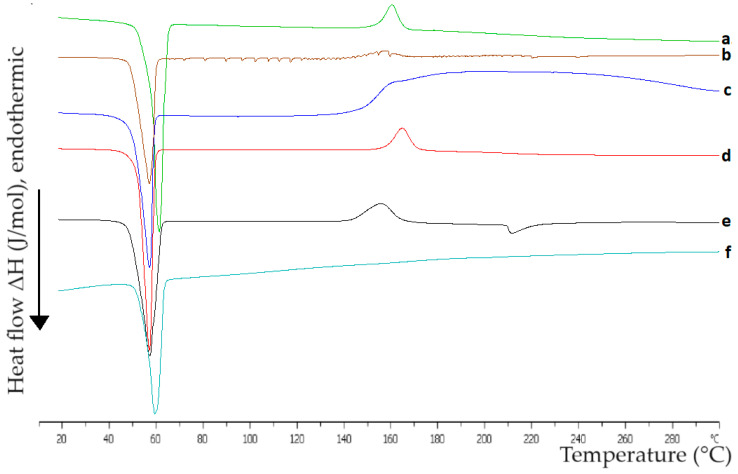
DSC thermograms. The heating curves of (**a**) P407, (**b**) P407/Tw80, (**c**) P407/MβCD, (**d**) P407/HPβCD, (**e**) P407/Tw80/MβCD, (**f**) P407/Tw80/HPβCD. The limits for the calculation of thermotropic parameters are from 10 °C to 300 °C.

A crucial aspect of developing hybrid systems composed of various materials lies in determining the interactions between them. Differential scanning calorimetry (DSC) serves as a valuable tool for analyzing these interactions. The samples examined were pure compounds of P407, Tw80, MβCD, and HPβCD, as well as their mixtures, including Tw80/MβCD at a 70:30 ratio, Tw80/HPβCD at a 70:30 ratio, P407/Tw80 at a 70:30 ratio, P407/MβCD at an 80:20 ratio, P407/HPβCD at an 80:20 ratio, and (P407/Tw80)/MβCD and (P407/Tw80)/HPβCD at an 80:20 ratio. The calorimetric heating profiles obtained by DSC analysis of pure compounds and composed systems in the solid state are displayed in Figure 2, and the associated calorimetric parameters are outlined in Table S1 (Supplementary Materials). It should be noted that other techniques like Fourier-transform infrared spectroscopy (FTIR) are also used to study the interactions between the materials of hybrid systems in the solid state. We used DSC analysis due to the highly reproducible phase transitions in a temperature range, which are of paramount importance for the performance of studies in the development of hybrid systems [44,45].

The wide endothermic peak at 60 °C for Tw80 (Supplementary Materials, Table S1) might align with Tw80’s cloud point at 65 °C, where the surfactant’s solubility in aqueous media significantly decreases. As a result, surfactant molecules aggregate, leading to phase separation and a cloudy appearance in the solution, as described by Pérez-González et al. [46] and Al-Sabagh et al. [47].

Regarding the DSC thermogram of pure MβCD, it exhibited one broad endothermic peak at 180 °C and another one at 75 °C, with a low value of enthalpy (Supplementary Materials, Table S1). In contrast, HPβCD’s DSC analysis showed an endothermic peak at 147 °C (Supplementary Materials, Table S1), possibly due to water loss from the crystal and the absence of a defined fusion event due to the lack of a crystalline structure [48]. When comparing the two β-CD derivatives, it was noted that MβCD presents a higher main transition temperature (T_m_) than HPβCD. Moreover, the pure compound of HPβCD exhibits a higher enthalpy value (Supplementary Materials, Table S1), indicating that the MβCD derivative possesses greater resistance to temperature increases and requires more energy to reach its melting point, potentially due to stronger intermolecular secondary interactions. CDs’ dehydration process is characterized by the endothermic peaks observed in the DSC thermograms [49]. Different studies have reported a wide range of peaks for MβCD [50,51,52,53] and HPβCD [54,55,56,57,58]. These variations could be attributed to differences in compound purity and sample preparation methods.

The DSC curves of P407, displayed in Figure 2, revealed a distinct, endothermic, symmetric peak at 57 °C, which corresponds to the melting point of the compound. This confirms that under these conditions, the substance is stable and does not undergo decomposition. These results are consistent with those reported in other studies [59,60,61,62,63]. In addition, a secondary exothermic peak was observed at 158 °C, which is in agreement with the findings of Pawar and Pande [64].

Regarding the P407/Tw80 mixture (Figure 2), it was observed that the main transition temperature was displaced at a lower temperature (53 °C) and there was a significant reduction of ΔH in comparison to the pure polymer (Supplementary Materials, Table S1), revealing interactions between P407 and the surfactant. The changes in thermal behavior observed in this mixture are driven by the amphiphilic characteristics of the materials, which enable them to interact with each other through hydrophilic and hydrophobic interactions. Specifically, the hydrophilic chains of P407 (i.e., PEO chains) interact with the head group of Tw80, while the hydrophobic PPO chains of the polymer interact with the hydrophobic subunits of the surfactant. The formation of some hydrogen bond or van der Waals interactions may also contribute to these changes. In line with previous studies [65], the decline in enthalpy values observed in a DSC curve, associated with a particular component following the addition of another material, is indicative of strong interactions between the two compounds.

Comparing the DSC results for the P407/MβCD and P407/HPβCD mixtures to those of pure P407, a slight shift of the main transition temperature to a lower one (53 °C) was noted. Moreover, the polymer’s exothermic peak was replaced with a much broader one at a higher temperature (188 °C) and one other peak at 162 °C with low enthalpy (9.85) appeared for the P407/MβCD and P407/HPβCD mixtures, respectively.

The DSC analysis of the P407/Tw80/MβCD mixture (as depicted in Figure 2) showed that the addition of MβCD to the binary system significantly impacted the polymer’s thermal behavior, as evidenced by the appearance of two new peaks: one exothermic peak at 153 °C and an endothermic peak with a low enthalpy at 210 °C. On the other hand, P407/Tw80/HPβCD displayed a completely different peak pattern since it caused only a slight displacement of the main transition to a higher temperature (55 °C). The observed differences in the DSC thermograms of the mixtures may be attributed to the interactions developed between the materials and may be indicative of the formation of an inclusion complex in the ternary systems. The development of an inclusion complex between polymers and CDs has also been proven by DSC analysis in other studies. Zhang et al. [66] reported the creation of an inclusion complex between the hydrophobic groups of the polymer upon their integration into the non-polar cavity of β-CD. Assembly formation was achieved through a combination of van der Waals forces and hydrophobic interactions during the inclusion complexation process.

The differences observed in mixtures with MβCD and HPβCD may be attributed to the dissimilar hydrophobic nature of the β-CD’s derivatives. The modifications at the β-CD substitution led to different interactions between the compounds and, subsequently, to different thermal behaviors. The DSC curves of the analyzed mixtures were characterized by low values of ΔΤ_1/2_ (half width at half peak height of the transition) (Supplementary Materials, Table S1), proving favorable cooperativity between the compounds.

Overall, the findings of the DSC analysis demonstrated the existence of a range of interactions between the compounds, and the presence of the non-ionic surfactant and CDs greatly changed the polymer’s thermal behavior, likely due to the creation of an inclusion complex.

The findings of our DSC studies are also confirmed by literature data provided by FTIR analyses investigating the spectra of either the pure substances or their combinations [67,68,69,70]. For example, FTIR analysis of pure P407 showed absorption bands at 3000–2850 cm^−1^, -CH_2_ stretch corresponding to the bonds present in PEO and PPO blocks, and stretching vibrations of C-C bonds at 1100 cm^−1^ and at 1380 cm^−1^ due to the symmetric angular deformation of CH_3_ [67]. Similar findings have been reported by Lin and Chang [68], who confirmed the interactions improved when P407 was combined with HPβCD. More specifically, a noticeable reduction in the intensity of the bands was noted, suggesting interactions between HPβCD and the copolymer, primarily due to van der Waals interactions. This observation led to the inference that P407 engages with the hydrophobic cavity of HPβCD. Also, Bin Jardan et al. [69] used FTIR analysis to identify the interactions developed between the materials of their ternary inclusion complex (comprising a pterostilbene, β-CD, and P407). Indeed, they proved that the combination of the materials diminished the peaks’ intensities and caused displacements of peaks compared to those of pure compounds. Finally, the higher affinity of β-CD to the PPO block of P407 was shown through the FTIR spectrum conducted by Di Donato et al. [70]. This extracted conclusion is supported by the observation that the characteristic bands of P407 spectra appeared with weakened intensity after the addition of high concentrations of β-CD.

### 2.2. Physicochemical Characterization of Systems in Aqueous Solutions—Evaluation of Their Biological and Physical Stability

On the first day of colloidal dispersion preparation (Section 3.2.2), measurements of particle size, the corresponding percentage of each population in each sample (weight of peak), polydispersity index (PDI), and zeta potential (z-potential) were carried out using dynamic and electrophoretic light scattering (DLS and ELS, respectively). The acquired data are presented in Table 1.

DLS analysis of the colloidal dispersion of P407 revealed the presence of a heterogeneous particle population. Specifically, three distinct populations were observed, exhibiting different average hydrodynamic radius (R_h_) values. The smallest one constitutes 6% of the total population, exhibiting an average R_h_ of 4 nm. The intermediate one, representing 38% of the total population, displays an average R_h_ of 39 nm and the largest one (55% of the total particles’ population) presents an average R_h_ of approximately 600 nm (Table 1). This indicates the presence of relatively large particles within the dispersion. In the literature, variations in the size of P407 particles have been reported [71]. The size of P407 particles can vary depending on various factors, including the concentration of the polymer, the method of preparation, and the source or manufacturing company. Upon the addition of the surfactant (Tw80), the particle size was reduced, resulting in a mean R_h_ of 29 nm. This reduction suggests that the surfactant had a dispersing effect on the larger particles, leading to the formation of smaller particles in the dispersion. However, despite the decrease in average particle size, our analysis uncovered significant heterogeneity among the particles, which is evident from the broad curves observed in Figure S1 (Supplementary Materials). Similar findings regarding the dispersing impact of the surfactant (Tw80) were previously documented by Kontogiannis et al. [72]. Their investigation focused on the alterations in the particle size of poloxamer 188 following the addition of Tw80. Their outcomes revealed a noteworthy reduction in the system’s particle size; however, there was also an increase in PDI value due to the presence of distinct populations of particles in the suspension.

The addition of CDs resulted in a reduction in particle size heterogenicity within the system, as demonstrated by the narrow curves observed in the size distribution diagram (Supplementary Materials, Figure S1). More specifically, upon the further addition of MβCD, the primary population of particles (corresponding to 97% of the total population in both hybrid systems) presented an average R_h_ equal to 104 nm, while the incorporation of HPβCD led to an R_h_ of 114 nm. Therefore, no significant distinctions in particle size were observed in ternary systems with these different β-CD derivatives. Furthermore, it was noted that the dominant population of ternary systems presented significantly decreased R_h_ values compared to those of the colloidal dispersion of P407 (Table 1), which is also supported by the findings of Li et al. [73]. They demonstrated that the presence of HPβCD could decrease the particle size of prepared polymeric nanoparticles and lead to the formation of nanoparticles with a uniform particulate distribution.

The ELS measurements of the created compositions in an aqueous solution showed a slightly negative surface potential (Table 1). Specifically, the surface charges of P407 and P407/Tw80/MβCD were measured to be −20.5 mV and −12.9 mV, respectively, which means that a slight electrostatic repulsion between the particles must exist. It is worth mentioning that the z-potential values of P407/Tw80 and P407/Tw80/HPβCD were found to be close to zero, indicating that the particle surfaces were mostly neutral in terms of charge. These particle size changes confirmed the results obtained by DSC experiments, which evidenced alterations in the DSC curves of the systems, proving the existence of interactions between the compounds utilized, which also modify the particle size of the coassembled systems in aqueous solutions.

The utilization of DLS has been widely employed to examine the characteristics and performance of nanostructures within serum media [74]. A mixture of fetal bovine serum (FBS) and phosphate buffer solution (PBS) at a weight ratio of 10:90 was used as a dispersion medium, simulating the physicochemical conditions of human plasma (Supplementary Materials, Table S2). The presence of proteins, such as albumin, in the biological medium influences the properties of the systems and this aspect is worth investigating [74]. In both ternary systems, the R_h_ of the systems’ main particle population increased and new populations emerged. As illustrated in Figure S2 (Supplementary Materials), the systems’ size distribution curves were broadened, indicating a high degree of polydispersity within the systems’ population, which is likely attributed to serum-induced aggregation. These results indicate the interactions between the system and plasma proteins improved. Based on the experimental findings mentioned above, it can be inferred that the hybrid nanostructures exhibit a certain degree of stealth characteristics and biological stability, attributed to the triblock P407 copolymer. The above results show that the P407 copolymer contains PEO chains, forming a hydrophilic corona which serves as a protective shield for the nanostructures against environmental factors. In a similar context, Chountoulesi et al. [74] demonstrated the corona effect and steric stabilization of P407, which effectively protected their nanosystems from the presence of albumin in FBS.

Additionally, the formulations did not show any significant alterations in size when exposed to conditions that mimic the pH and temperature conditions of the human nasal cavity. Specifically, at a pH of 5.6 and a temperature of 34 °C, the systems retained their initial dimensions without observable modifications (Supplementary Materials, Table S2). These findings strongly suggest that the formulations remain structurally intact and exhibit stability when exposed to simulated nasal cavity conditions, whereas the PEO chains partially impart biological stability to the composed structures.

The ternary systems’ stability was also evaluated by performing DLS measurements on dispersions that had been stored in glass vials at 4 °C in the refrigerator over a period of 30 days. This temperature is well-established for its ability to slow degradation processes and maintain colloidal integrity, making it a common choice in the determination of the kinetic stability of prepared systems [71,75,76,77]. As depicted in Figure S3 (Supplementary Materials), the systems remained stable during this period as their R_h_ did not change significantly. As previously noted, the PEO chains of P407 extend beyond the system, providing steric stabilization due to the hydrophilic corona of PEO chains [74]. Additionally, according to Akbar et al. [78], the hydrophobic PPO chains of P407 align with the non-aqueous domains of the systems. Simultaneously, the hydrophilic component of P407 (PEO chains) extends outward into the aqueous environment. This extension of the PEO block from the particle surface effectively prevents particle aggregation and avoids sedimentation phenomena.

It was postulated that the presence of electrostatic interactions between the particles in the P407/Tw80/MβCD hybrid system may have been responsible for the physical stability of the system, in addition to the property of steric repulsion provided by the hydrophilic corona of PEO chains [79]. Indeed, the results obtained from the DLS experiments confirm this hypothesis.

### 2.3. Study of the Internal Hydrophilic/Hydrophobic Environment of the Hybrid Systems

Fluorescence spectroscopy was employed to gain insight into the internal structure of the prepared systems, utilizing pyrene as a hydrophobic probe, which has the capability of being encapsulated within the hydrophobic domains of amphiphilic polymeric structures formed in aqueous solutions. The ratio of intensities between the first and the third peak in the pyrene emission spectrum, I_1_/I_3_, serves as a sensitive measure of the polarity of the environment surrounding the probe. As outlined in the literature [80], I_1_/I_3_ ratio values between 1 to 1.3 indicate the presence of a hydrophobic microenvironment, while values in the range of 1.7 to 1.9 typically correspond to a polar environment.

P407 is one of the most widely used thermoresponsive polymers [81]. As such, it was deemed appropriate to conduct fluorescence spectroscopy experiments under varying temperature conditions, including room temperature (25 °C), human body temperature (37 °C), and a temperature of an abnormal condition (50 °C) [74,82]. The results are presented in Table 2.

The results indicated that the addition of the surfactant did not have a significant impact on the micropolarity of the systems at 37 °C and 50 °C. However, at 25 °C, the micropolarity of the systems decreased due to interactions between the amphiphilic compounds. Moreover, the addition of β-CD derivatives increased the hydrophilicity of the systems, as evidenced by the increase in I_1_/I_3_ values attributed to the hydrophilic outer surfaces of CDs. Specifically, the P407/Tw80/MβCD system exhibited an elevated I_1_/I_3_ value at 25 °C and 37 °C and an almost unaffected I_1_/I_3_ value at 50 °C. Lastly, the incorporation of HPβCD caused a rise in hydrophilicity, most prominently at 50 °C. The temperature shift from 25 °C to 37 °C resulted in an increase in I_1_/I_3_ value, indicating a heightened hydrophilicity of the systems. The results showed that the highest increase in I_1_/I_3_ value occurred in the P407/Tw80 system. Indeed, at a temperature of 25 °C, the I_1_/I_3_ value was 1.19, whereas at a temperature of 37 °C, it increased to 1.31. On the other hand, the increase in temperature from 37 °C to 50 °C led to a decrease in I_1_/I_3_ value, indicating a shift towards increased hydrophobicity, except for the P407/Tw80/HPβCD system, which remained constant. Fluorescence spectroscopy demonstrated that P407/Tw80/MβCD was particularly sensitive to this change in temperature, as the I_1_/I_3_ value reduced from 1.35 (37 °C) to 1.20 (50 °C). Presumably, some reorganization of the components takes place in hybrid coassembled aggregates at higher temperatures.

These findings are consistent with the data obtained from DSC experiments. The DSC results showed that all systems containing P407 exhibited a main transition peak around 50 °C, indicating a change in their thermal behavior and hydrophobicity/hydrophilicity ratio at this temperature. Therefore, the results obtained from the fluorescence spectroscopy technique confirm that P407 is a thermoresponsive polymer whose properties are influenced by temperature. In turn, this affects the internal environment of the hybrid systems.

### 2.4. Thermal Characterization of Colloidal Dispersions in Aqueous Solutions and Surface Tensiometric Analysis

**Figure 3:**
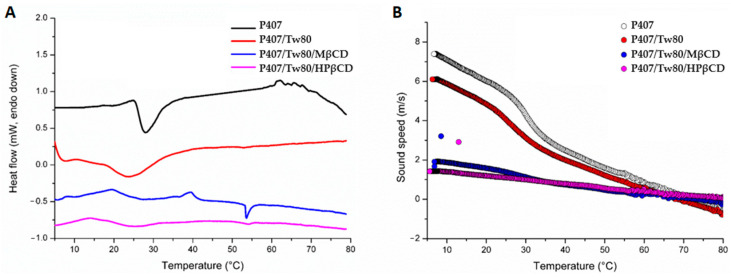
(**A**) mDSC traces and (**B**) sound speed vs. temperature profiles from HR-US for P407, P407/Tw80, P407/Tw80/MβCD, and P407/Tw80/HPβCD formulations.

Figure 3A and Table 3 depict the microcalorimetry (mDSC) and high-resolution ultrasound spectroscopy (HR-US) thermograms and thermodynamic parameters of systems containing P407 with or without the presence of Tw80, MβCD, and HPβCD, respectively.

A clear endothermic transition was observed at 28 °C in the thermograms of P407, which is recognized as its main transition temperature. The addition of Tween 80 markedly affected the thermal behavior of the polymer, as evidenced by a broader peak with a higher enthalpy (ΔH_P407/Tw80/MβCD_ = 0.220 J/g) and centered at around 24 °C. These changes indicate the presence of interactions between the polymer and the surfactant, driven by the amphiphilic nature of the compounds, enabling the formation of both hydrophilic and hydrophobic interactions. Furthermore, the presence of MβCD and HPβCD in the P407/Tw80 system led to a slight displacement of the main transition temperature to lower values and the appearance of broader peaks, as seen from the remarkable reduction in enthalpy (ΔH_P407/Tw80/MβCD_ = 0.116 J/g, ΔH_P407/Tw80/MβCD_ = 0.101 J/g) in comparison to that of the P407 sample (ΔH_P407_ = 0.175 J/g) (Table 3). This observation could be attributed to the solubilization of the system due to the possible complexation of the compounds. It should be noted that the slight differences observed in the thermal behavior of the ternary systems with MβCD and HPβCD in the dispersion state may be due to the structures of CDs and their different water solubilities. The results of mDSC analysis revealed the presence of diverse interactions among the compounds of the system, which may be a result of self-assembly processes among P407, Tw80, and CDs.

To verify the main transitions observed through mDSC, a supplementary methodology known as HR-US was utilized (as represented in Figure 3B). The analysis of the data obtained from HR-US, measuring the variation in sound speed over temperature, revealed that the transition temperatures for all systems ranged from 27 °C to 30 °C, highlighting again the effect of the presence of Tw80, MβCD, and HPβCD on the main thermal transition of P407.

The observations from the above techniques indicated that there are dissimilarities in the thermotropic characteristics of the prepared systems when compared to those obtained by DSC. These differences can be attributed to variations in the heating rates employed in the methods and the different states of the systems examined. In DSC, the interactions between the pure compounds were studied in the solid state. In contrast, the mDSC and HR-US techniques were utilized to analyze the particles in the dispersion state, where self-assembly or coassembly processes could potentially have taken place. Consequently, the presence of steric interactions resulting from the hydrophilic PEO chains led to the formation of colloidal particles exhibiting distinct thermal behavior in the dispersed liquid state.

Surface tension (γ) is a measure of the effectiveness of a surfactant in decreasing air–water surface tension [83]. The calculated surface tension values for the prepared systems are presented in Table 3. The value of γ for P407 was found to be 39 mN/m. The addition of Tween 80, as well as the further incorporation of HPβCD, did not have a significant impact on the value of γ for P407. With regards to the addition of MβCD, the surface tension values for the system remained largely constant and did not appear to be directly affected by the presence of MβCD. Overall, the results of the tensiometric analysis demonstrated that the presence of the surfactant and β-CD derivatives did not cause a significant alteration in γ values for P407-containing hybrid systems.

### 2.5. Morphological Characterization of Hybrid Systems

**Figure 4:**
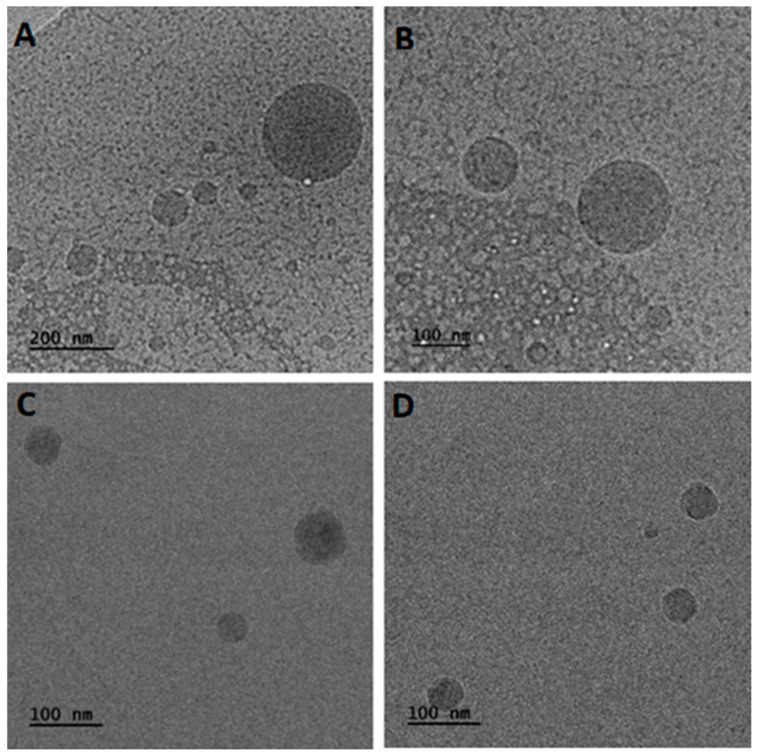
Cryo-TEM micrographs of hybrid P407/Tw80/MβCD (**A**,**B**) and P407/Tw80/HPβCD (**C**,**D**) systems.

The morphological characteristics and dimensions of the prepared hybrid structures were also investigated using cryogenic transmission electron microscopy (cryo-TEM) images. The micrographs obtained from the analysis reveal the presence of spherical particles within the ternary systems (Figure 4).

Specifically, the P407/Tw80/MβCD system exhibited particles with sizes ranging from 30 to 250 nm. The size of spherical particles in the P407/Tw80/HPβCD system was found to be 40–115 nm. Furthermore, it is imperative to take into consideration that the systems’ colloidal concentration (10 mg/mL) may influence the particles’ structural morphology. The observed shape asymmetry could also have arisen from the heterogeneous incorporation of triblock copolymer chains within the polymer/surfactant/CD inclusion complex. The results of the DSC, mDSC, and HR-US analyses demonstrated a change in the materials’ thermal behavior and the possible formation of an inclusion complex comprising the polymer, surfactant, and CD, with strong interactions observed between these components, likely due to the hydrophobic interior and hydrophilic exterior of the inclusion complex. This complex is illustrated as the spherical particle observed in cryo-TEM images.

The structures visualized in cryo-TEM images present differences in the average R_h_ values of the samples determined by intensity-weighted measurements in DLS. Besides the fact that both techniques are used to study particle size in structures, they are based on fundamentally different principles, which can lead to differences in the particle size measurements they provide. The cryo-TEM technique offers the advantage of investigating colloids directly in the vitrified, frozen-hydrated state following the plunge freezing of dispersions. This unique approach closely resembles the colloids’ native state, enabling the revelation of valuable insights into their internal and three-dimensional structure [84]. In contrast, DLS measures the particle size of hybrid colloidal dispersions in the solution state in which self-assembly phenomena take place. It should be noted that there are some differences in the average size of nanoparticulate systems between DLS and cryo-TEM [85,86]. These differences are strongly associated with the solution state of the matter in DLS and the interactions of each particle with its neighboring particles (attractive or repulsive interactions). In other words, this discrepancy is also attributed to the swelling and Brownian motion of nanoparticles in the dispersion medium during DLS measurements, whereas TEM observations reflect the diameter of the solid state of the particles.

To conclude, cryo-TEM examines colloids in a frozen-hydrated state after plunge freezing, while DLS investigates the particle size of systems in a solution state. Particle sizes assessed by these two methods vary due to differences in their underlying principles [85,86].

### 2.6. Cytotoxicity of Colloidal Dispersions

**Figure 5:**
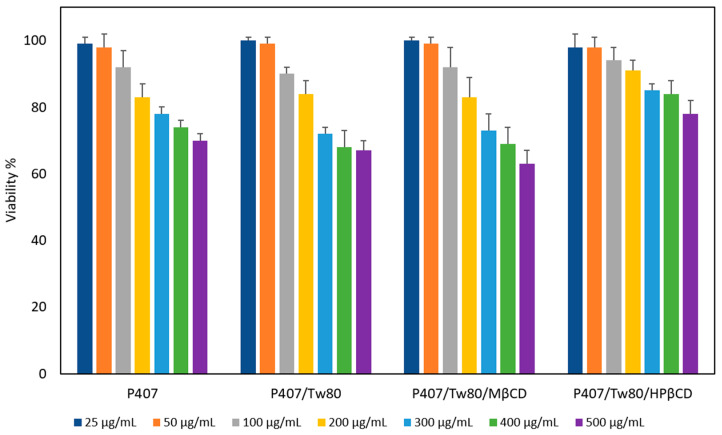
MTΤ cell viability assay after 24 h treatment of HEK-293 of hybrid colloidal systems: pure P407, P407/Tw80, P407/Tw80/MβCD, and P407/Tw80/HPβCD systems. Cell viability is expressed as % cell viability ± SD between two experiments.

The toxicity profiles of all the prepared systems were evaluated and they are presented in Figure 5. Cell viability was expressed as a percentage (%) of cell viability ± standard deviation (SD) between two experiments. The results then underwent statistical analysis, as described below in Section 3.2.15. The analysis of the data revealed that all systems displayed high cell viability at lower concentrations (25, 50, and 100 μg/mL), with values exceeding 90%. As the concentration of the systems increased, cell viability exhibited a proportional decrease, reaching 63–70% at the highest tested concentration of 500 µg/mL.

It is noteworthy that the inclusion of HPβCD had a positive impact on the toxicity profile of the prepared hybrid systems. Specifically, the ternary system containing HPβCD exhibited the highest cell viability at the highest concentrations tested (84% and 78% at concentrations of 400 and 500 µg/mL, respectively). The superior cell viability of P407/Tw80/HPβCD is statistically significant at concentrations of 300 and 500 µg/mL, compared to either P407 alone or in combination with Tw80 [*p* < 0.05, 95% confidence interval (CI)], as well as to the ternary systems containing MβCD (*p* < 0.05, 95% CI). In contrast, the presence of MβCD did not have any impact on cell viability compared to either P407 alone (*p* > 0.05, 95% CI) or in combination with Tw80 (*p* > 0.05, 95% CI), resulting in decreased cell viability, with the lowest observed value (63%) at a concentration of 500 µg/mL. It is known that nanotoxicity is influenced by multiple parameters, such as size and shape, which appear to be major contributors to cell internalization and cytotoxicity [87].

Overall, the results of the MTT assay revealed that at low concentrations (25–200 μg/mL), all these combinations can be considered biocompatible, since the exhibited cell viability higher than 80%. Notably, the hybrid system containing HPβCD was also deemed biocompatible, even at higher concentrations (300 and 400 μg/mL). However, as the dose was increased, a correlation between dose and cytotoxicity was observed, with the degree of cytotoxicity varying based on the materials’ composition. As a result, it was found that all systems displayed dose-dependent and material-dependent toxicity on HEK293 cell lines. The findings from these toxicity studies will serve as a guide for evaluating the prepared systems as carriers for drug delivery.

### 2.7. Preformulation Studies and Physicochemical Characterization of RH-Containing Systems

**Figure 6:**
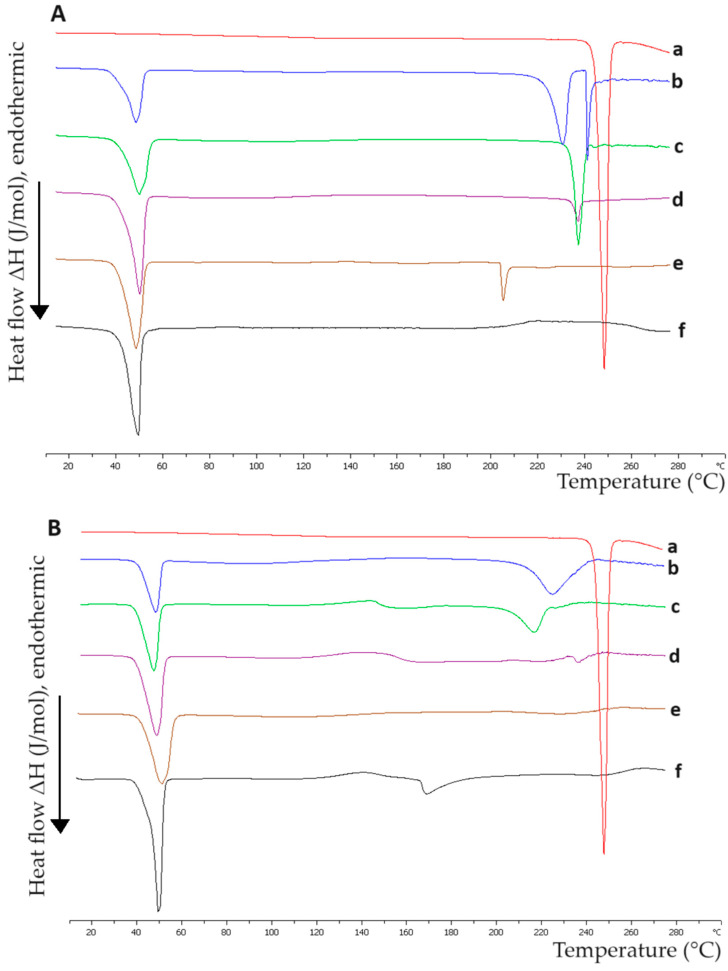
DSC thermograms. Heating curves of (**A**) (**a**) RH and (P407/Tw80/MβCD)/RH at different weight ratios: (**b**) 10:10, (**c**) 10:5, (**d**) 10:1, (**e**) 10:0.5, (**f**) 10:0.1. Heating curves of (**B**) (**a**) RH and (P407/Tw80/HPβCD)/RH at different weight ratios: (**b**) 10:10, (**c**) 10:5, (**d**) 10:1, (**e**) 10:0.5, (**f**) 10:0.1. The limits for the calculation of thermotropic parameters are from 10 °C to 300 °C.

RH served as the model drug for investigating the loading and release properties of the prepared hybrid polymer/surfactant/CD systems. The results taken from the DSC analysis for the P407/Tw80/MβCD/RH and P407/Tw80/HPβCD/RH formulations with varying concentrations of the API are presented in Figure 6 and Table S3 (Supplementary Materials).

Regarding the DSC results obtained for the pure RH compound, a sharp endothermic peak was observed at 249 °C, with a high value of enthalpy (ΔH = −22.69 KJ mol^−1^). This peak is indicative of RH’s melting point and serves as evidence of its high crystallinity, which is consistent with other studies [88,89,90]. As we mentioned above, the thermogram of the P407/Tw80/MβCD system displayed a main intense endothermic peak at 53 °C—which may be attributed to the crystalline regions of the PEO polymer and corresponds to the melting point of P407—as well as a secondary exothermic peak at 153 °C and a ternary endothermic peak at 210 °C with a low value of enthalpy (ΔH = −1.64 KJ mol^−1^). The DSC scan of the P407/Tw80/HPβCD system revealed a main intense endothermic peak at 55 °C with ΔH value equal to −32.29 KJ mol^−1^.

According to the results of DSC experiments for P407/Tw80/MβCD with RH, the main transition temperature of P407 remained consistently stable at 49–50 °C across all weight ratios. However, with an increase in the weight ratio of RH, a reduction in the enthalpy value of the endothermic peak corresponding to P407 was observed. This finding suggests that the presence of the API may induce alterations in the thermotropic behavior of the P407/Tw80/MβCD hybrid system. Thus, the reduction in the enthalpy value as the system-to-RH weight ratio increases proves the interactions developed between the system and RH. Lazaratos et al. [65] reported similar observations. Notably, the diminished enthalpy value in the DSC curve of dipalmitoylphosphatidylcholine, as the amount of polyarabic acid increased, suggested a range of hydration forces and interactions between the polysaccharide chains of the nanoparticles and the polar head groups of the dipalmitoylphosphatidylcholine lipid bilayer.

RH’s endothermic peak changed as a function of increasing weight ratios of the drug. Moreover, as the system (P407/Tw80/CD) ratio in the formulation was increased, the sharpness of the drug’s endothermic peak was decreased. A displacement of RH’s transition temperature to lower values was also observed in all ratios, varying from 12 °C to 44 °C, as depicted in Figure 6 and Table S3 (Supplementary Materials). This alteration indicated a partial loss of crystallinity observed in RH upon its integration into the hybrid complex, a finding supported by Hussein et al. [90]. A displacement of its transition temperature to lower values was observed in all ratios, varying from 12 °C to 44 °C. At the lowest concentration of the drug, the peak of RH was converted into a broadened exothermic peak at 221 °C, which was also proven by the extremely high ΔT_1/2_ value (50.62 °C) (Supplementary Materials, Table S3). This may indicate that the drug was uniformly dispersed at the molecular level within the hybrid system [90] or that its concentration was too low to be detected by DSC. The highest shift of the drug’s peak was observed at a weight ratio of system/RH equal to 10:0.5. The corresponding thermogram presented an endothermic peak at 205 °C with a significantly reduced enthalpy value (ΔH = −1.42 KJ mol^−1^). At weight ratios of system/RH of 10:1 and 10:5, the drug’s transition temperatures were decreased by 12–15 °C (T_m_ = 234 °C and 237 °C, respectively), whereas ΔH values were reduced to −1.48 KJ mol^−1^ and −7.49 KJ mol^−1^. The ratio equal to 10:1 showed excellent cooperativity of the materials, as inferred from the low value of ΔT_1/2_ (2.28 °C). Upon DSC analysis of the system with the highest RH concentration, a notable transformation of the main endothermic peak was observed, resulting in the presence of two new endothermic peaks at 231 °C and 241 °C, exhibiting ΔH values of −9.12 KJ mol^−1^ and −3.17 KJ mol^−1^, respectively. These observations suggest that the system may have reached its maximum capacity for drug incorporation at a weight ratio of 10:1.

DSC analyses of P407/Tw80/HPβCD with RH revealed a minor downward shift in the primary transition temperature, with a decrease of 5–7 °C observed across all weight ratios. However, with an increase in the weight ratio of RH, a reduction in the enthalpy value of the main endothermic peak was observed. These findings suggest that the presence of the API may have induced alterations in the thermotropic behavior of the P407/Tw80/HPβCD hybrid system. Notably, at the lowest concentration of the drug (system-to-RH weight ratio equal to 10:0.1), a new endothermic peak, characterized by T_m_ = 170 °C and ΔH value equal to −4.24, emerged. Likewise, as with P407/Tw80/MβCD, this suggests that the drug was uniformly distributed at the molecular level within the hybrid system, or its concentration was too low to be detected efficiently by DSC. At a weight ratio of 10:0.5, no discernible curves associated with RH were identified, likely due to the extremely low drug concentration, which fell below the detectable limit of DSC. At system-to-RH weight ratios of 10:1, 10:5, and 10:10, the drug’s transition temperature decreased by 11 °C to 32 °C (T_m_ equal to 238 °C, 217 °C, and 226 °C, respectively), while the enthalpy value increased in line with the concentration of RH.

When comparing interactions in RH ternary systems utilizing different derivatives of β-CD, it is evident that, in both cases, an increase in the concentration of RH resulted in elevated ΔH values for the RH curve. Simultaneously, the corresponding enthalpy value of the P407 curve diminished. Additionally, at low concentrations of RH, the absence of any discernible curve corresponding to the drug may be attributed to the uniform molecular distribution of the drug within the hybrid system or a concentration too low to be efficiently detected by DSC. The cooperativity between materials is notably enhanced at weight ratios of system/RH of 10:1 and 10:5. Moreover, RH systems with MβCD exhibit superior cooperativity between compounds, as evidenced by lower ΔT1/2 values compared to the corresponding ones in systems with HPβCD.

Overall, based on the results of the DSC experiments, a multitude of interactions arose between the hybrid systems and the drug. In our opinion, the most promising weight ratios for further investigation are 10:1 and 10:5, as they demonstrated superior cooperativity between the materials, as evidenced by the lower values of ΔT_1/2_ observed in all peaks in comparison with the other weight ratios (especially for the P407/Tw80/MβCD/RH system), as well as the fact that at these weight ratios, the highest incorporation of the drug was achieved.

For this reason, hybrid ternary systems with RH at weight ratios of 10:1 and 10:5 were prepared by the thin-film hydration method (Section 3.2.2). DLS measurements were conducted on the day of their preparation and the results are presented in Table 4.

In the case of (P407/Tw80/MβCD)/RH at the weight ratio of 10:1, the results revealed a dominant population of 92% with an R_h_ of around 99 nm. Additionally, a minor population (7% of the total particle population) with an R_h_ of 4 nm was observed. Regarding the weight ratio of 10:5, DLS analysis showed a different particle distribution, revealing a population with an R_h_ of 211 nm (80% of the total particle population) and another with an R_h_ of 8 nm (20% of the total particle population). The addition of RH led to an increase in particle heterogeneity, as illustrated by the broad curves shown in Figure S4 (Supplementary Materials) in both cases. These results suggest that the specific weight ratios of the components in the system influence particle size distribution, potentially due to different interactions and coassembly behaviors among the components. These outcomes align with the findings of Kuplennik and Sosnik [29], who showed that variations in system-to-API weight ratio can either increase or decrease the particle size and PDI value of fabricated nanoparticles.

**Figure 7:**
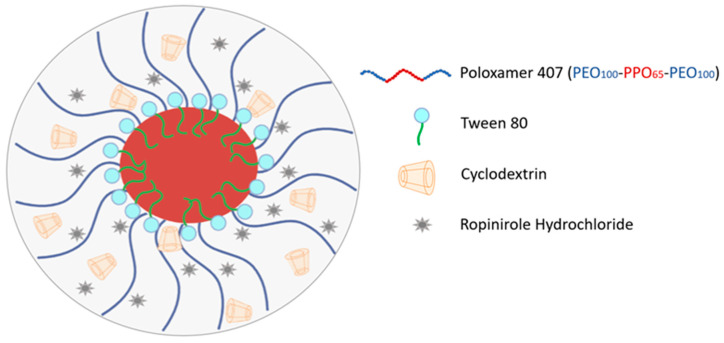
Illustration of the RH systems based on the results obtained from the applied techniques.

The P407/Tw80/HPβCD/RH systems comprised two populations. The smaller population, constituting 12% and 20% of the total, demonstrated an R_h_ of 6 nm in the systems with weight ratios of (P407/Tw80/HPβCD)/RH of 10:1 and 10:5 *w*/*w*, respectively. The dominant population, representing 88% and 80% of the total in the corresponding systems, exhibited an R_h_ of 60 nm. The addition of RH to the P407/Tw80/HPβCD system resulted in a reduction in the particle size of both populations, accompanied by an augmentation in the system’s heterogeneity. This increase in heterogeneity was evident in the rise in the PDI value, as evidenced by the broadened curves illustrated in the DLS diagrams (Supplementary Materials, Figure S4B). As a result, the two distinct β-CD derivatives facilitated a range of diverse interactions, leading to the formation of various bonds within the system and the RH. Consequently, this disparity ultimately contributed to differing effects on the particle size of the systems. Figure 7 illustrates a potential configuration of the RH systems, based on the results obtained from all the applied techniques.

### 2.8. RH Content in the Prepared Systems

RH content ranged from 104.06% to 106.08% (0.052 to 0.053 mg of RH, respectively) of the theoretical loading dose (observed differences less than 10%), and standard deviations varied from 0.92% to 2.44%.

### 2.9. RH’s Release from the Hybrid Formulations by In Vitro Diffusion Experiments

In vitro release experiments of RH from the prepared formulations were performed using diffusion Franz cells and regenerated cellulose membranes with defined pore sizes as diffusion barrier. A molecular mass cut-off of 1000 Da enabled the permeation of free RH, blocking the excipients and/or the possible derived complexes in case of interactions occurring among the components of each formulation. The amount diffused is expressed as a percentage of the loading dose. RH is a freely soluble compound, and therefore, no differences were expected between the release profiles of the prepared formulations.

**Figure 8:**
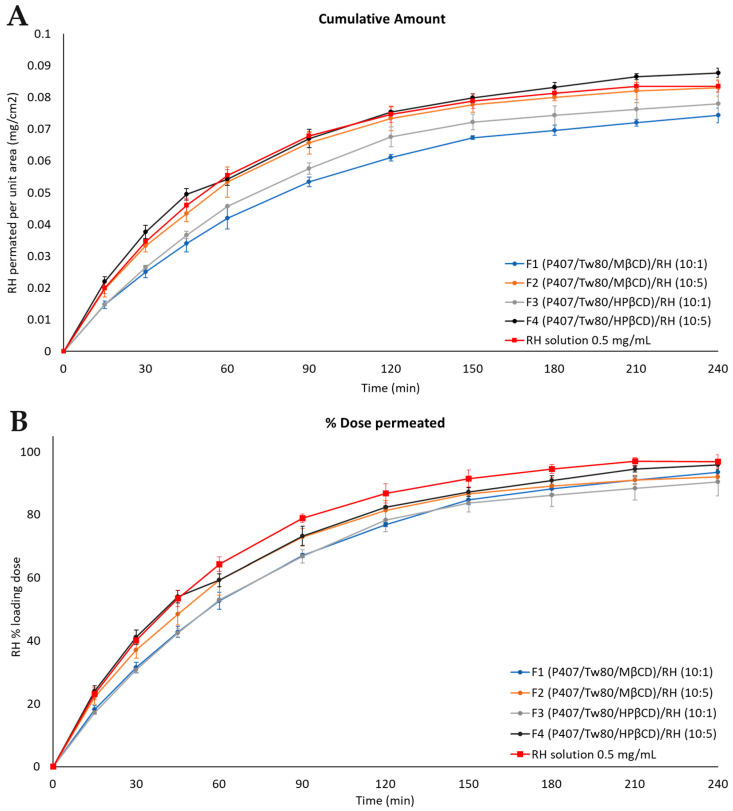
Permeation profiles of RH P407 ternary systems through regenerated cellulose membranes for colloidal dispersions at weight ratios of 10:1 and 10:5 in comparison to RH solution (0.5 mg/mL). Results are expressed as (**A**) quantity permeated per unit area (mean ± SD, *n* = 3) and (**B**) % loading dose permeated for the tested formulation (mean ± SD, *n* = 3).

Figure 8 presents the time profiles of the cumulative amount of permeated RH per unit area and the amount of permeated RH as a percentage of the loading dose for the prepared formulations and the corresponding control solution with a concentration of 0.5 mg/mL. Table 5 provides information on the percentage (%) of RH loading dose permeated for the tested formulations, the mass balance of RH in each formulation, and the percentage (%) of the RH loading dose retained by the cellulose membrane. All samples were analyzed using high-performance liquid chromatography (HPLC)-PDA (Section 3.2.13).

No significant differences [*p* > 0.05, 95% CI among the same time points] were observed between formulations with the same weight ratios of the components and different β-CD derivatives (F1 vs. F3 and F2 vs. F4).

Regarding the percentage of the loading dose permeated over time, it exhibited significant variations among formulations with different concentrations of RH during the experiment. Formulations F2 and F4 (containing either MβCD or HPβCD, respectively, and an RH concentration of 5 mg/mL), demonstrated a more pronounced release profile of the drug compared to formulations F1 and F3, which contained either MβCD or HPβCD, respectively, and an RH concentration of 1 mg/mL. The most substantial differences (*p* < 0.05, 95% CI) were observed between formulations F3 and F4 from the beginning of the experiment up to 45 min.

When comparing the prepared formulations with the control solution (0.5 mg/mL, PBS pH = 5.6), it is noteworthy that the release profile of F4 and F2 closely resembles that of the RH solution. Notably, F4 displayed the highest cumulative amount and release profile among all colloidal dispersions throughout the entire duration of the experiment. Statistical analysis revealed no significant differences (*p* > 0.05, 95% CI) for formulations F4 and F2 compared to the control RH solution (0.5 mg/mL, PBS pH = 5.6). However, formulations F1 and F3 (containing MβCD and HPβCD, respectively, and an RH concentration of 1 mg/mL) exhibited statistically significantly lower release profiles than the control solution (0.5 mg/mL, PBS pH = 5.6) (*p* < 0.05, 95% CI).

By utilizing Equation (1), the flux (J) values for the manufactured formulations were calculated and ranged from 4.9 × 10^−4^ ± 5.1 × 10^−5^ to 5.9 × 10^−4^ ± 8.9 × 10^−5^ μg/cm^2^/min (Supplementary Materials, Table S4). Additionally, the R-square values ranged from 0.8978 ± 0.0092 to 0.9572 ± 0.0054, revealing a strong correlation between the concentration of the substance and the rate of release, which is consistent with the characteristics of a first-order reaction (Supplementary Materials, Table S4). Furthermore, the quantification of retained RH content within the cellulose membrane fell within the range of 0.37 ± 0.02 to 0.50 ± 0.07 of the loading doses.

Overall, the prepared hybrid RH systems exhibit a remarkably high mass balance of up to 90% in all cases (Figure 8, Table 5). The most rapid increase in release rate was achieved at a concentration of RH equal to 5 mg/mL, indicating that different weight ratios of the components influence the rate and extent of drug release. Notably, the most beneficial release profile, observed in formulations F2 and F4 (system/RH weight ratio equal to 10:5), can be attributed to the improved interactions among the components of each formulation, effectively facilitating the diffusion of the drug into the membrane.

To conclude, as RH is classified as a BCS class III drug, presenting high water solubility, findings from the in vitro experiments did not permit the identification of crucial differences among the release profiles of the formulations, demonstrating a remarkably high mass balance of up to 90% in all cases. Therefore, it can be concluded that the presence of particles did not significantly affect the solubility of RH compared to the bulk form of the drug. Additional investigation involving ex vivo permeation experiments is needed to corroborate these findings.

### 2.10. RH’s Ex Vivo Mucosal Permeation from the Hybrid Formulations

**Figure 9:**
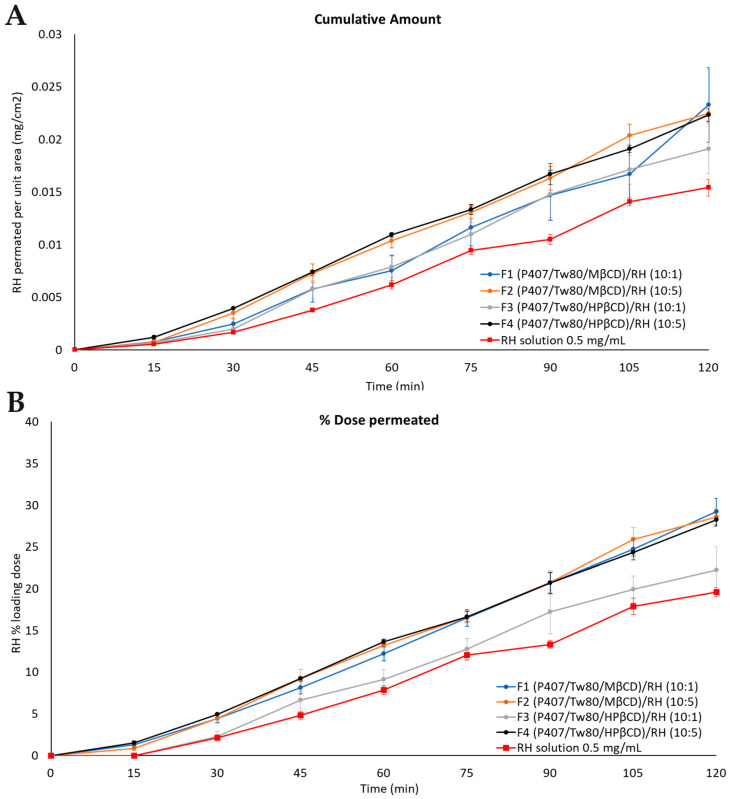
Permeation profiles for colloidal dispersions of RH P407 ternary systems through rabbit nasal mucosa for formulations at weight ratios of 10:1 and 10:5 in comparison to RH solution (0.5 mg/mL). Results are expressed as (**A**) quantity permeated per unit area (mean ± SEM, *n* = 4) and (**B**) % loading dose permeated for the tested formulation (mean ± SEM, *n* = 4).

The results of ex vivo permeation experiments are presented in Figure 9. The quantification of RH remaining in the nasal mucosa barrier revealed that 17.44 ± 2.12% of the loading dose was retained on average by the biological membrane. The membrane retention data of each formulation expressed as the percentage (%) of the loading dose retained by the nasal mucosa barrier ± standard error of the mean (SEM) are included in Table 6.

The RH solution (0.5 mg/mL, PBS pH = 5.6) presented a percentage of the loading dose permeated through the nasal mucosa equal to 19.62 ± 0.53. Formulations F1, F2, and F4 enhanced the permeability of RH up to 50% compared to the RH solution. In contrast, formulation F3 displayed a less favorable permeation profile, closely resembling that of the RH solution. Formulations F2 and F4, with a hybrid-system-to-RH weight ratio of 10:5, demonstrated a notably enhanced profile, as depicted in Figure 9. No significant differences (*p* > 0.05, 95% CI) were observed between them, suggesting that the two CD derivatives did not exert a differential effect on permeation through the nasal mucosa.

The J across the nasal mucosa barrier for all the tested formulations varied from 1.7 × 10^−4^ ± 1.0 × 10^−5^ to 2.0 × 10^−4^ ± 1.0 × 10^−5^ (μg/cm^2^/min), while the apparent permeability (*P_app_*) ranged from 0.35 to 0.40 (cm/min). The values of J and *P_app_* for each formulation are included in Table S4 (Supplementary Materials).

It has been reported that P407 can enhance drug transport through the nasal mucosa by decreasing the viscosity and elasticity of the mucus [91,92,93]. Saha et al. [93] developed a nanosuspension stabilized with P407, incorporating rotigotine for intranasal administration. The study of the in vitro dissolution of the optimized formulation demonstrated a rapid dissolution rate, with 95% of the cumulative drug dissolving within the first 15 min. The rotigotine nanosuspension exhibited a 20-fold increase in nasal permeation compared to a pure drug suspension, using goat nasal mucosa as a diffusion barrier. In addition, the intranasal administration in mice of an optimized zotepine formulation created by Pailla et al. [92], comprising P407 (0.3% *w/v),* hydroxypropyl methyl cellulose E15 (0.3% *w/v*), and soya lecithin (0.4% *w/v*), exhibited significantly elevated brain concentrations, with an 8.6-fold increase and a 10.79-fold rise in AUC_0–24h_ compared to intravenous zotepine solution.

Lin et al. [13] previously reported a synergistic effect between P407 and CDs. Their study focused on the intranasal delivery of an antiparkinsonian drug using a thermosensitive gel formulation containing P407 (20%), poloxamer 188 (1%), PEG-6000 (1%), and HPβCD (3%). Pharmacokinetic analysis revealed a noteworthy 1.6-fold increase in drug bioavailability and a 2.1-fold increase in brain targeting compared to oral administration. This highlights the potential of combining polymers, particularly P407 in the highest proportion, with CDs to enhance drug bioavailability following intranasal administration.

Therefore, the materials of our system, namely P407, Tw80, and CDs, serve as permeation enhancers by interacting with the mucosal epithelium, thereby influencing the integrity of the barrier and ultimately enhancing RH permeability. This suggests a positive impact resulting from the synergistic interactions of the three components.

### 2.11. Comparison of Polymer/Surfactant/Cyclodextrin System with other Hybrid Formulations for Nose-to-Brain Delivery of RH

The effectiveness of nose-to-brain RH delivery has been given increasing attention recently. Several types of hybrid nanoparticles have already been developed for the nasal administration of RH [94,95,96,97]. In all cases, the added value of these systems comes from the combination of materials of a different nature, i.e., polymers, CDs, or lipids, which enhances the permeability of RH, leading to site-specific targeting as well as controlled release. From a technological point of view, different types of nanoparticles, ranging from mucoadhesive ones to nanocomplexes, can lead to superior biological behavior, too [94,95,96,97].

More specifically, Chatzitaki et al. [94] fabricated mucoadhesive nanoparticles by combining poly (lactic-co-glycolic acid) and chitosan. These nanoparticles were used for the encapsulation of RH and demonstrated the ability to enhance the apparent ex vivo permeability of RH across sheep nasal mucosa by 3.22 times.

An improvement in RH nasal membrane permeability through sheep nasal mucosa was also reported by Pardeshi and Belgamwar [95], who fabricated a nanoplex by using dextran sulfate sodium. RH exhibited a controlled release pattern from the nanoplex, reaching a cumulative release of 44.98% ± 1.7% over the 24 h experimental duration.

Additionally, Jafarieh et al. [96] formulated a polymeric nanoparticulate carrier consisting of chitosan nanoparticles containing RH to facilitate targeted delivery to the brain. The in vitro release pattern of RH from the formulation demonstrated an initial rapid release of around 35% within 1 h, followed by a gradual and sustained drug release over 18 h, achieving a cumulative release of 89.18 ± 2.43%. The ex vivo experiments, using porcine nasal mucosa as a permeation barrier, revealed at least a twofold increase in the amount of permeated RH for the RH NPs compared to the control RH solution.

To the same context, Pardeshi et al. [97] also designed and fabricated polymer–lipid hybrid nanoparticles. Their in vitro release study showed a sustained release profile of RH from the tested formulation, whereas the percentage of the drug permeated across sheep nasal mucosa after 8 h was found to be 69.88 ± 1.48%. In vivo pharmacodynamic studies confirmed that the tested nasal formulation exhibited comparable therapeutic activity to the oral formulation, demonstrating effectiveness at a lower dosage. This suggests the potential to reduce both dose and dosing frequency and maximize the therapeutic index of RH.

Considering the above hybrid systems that were recently developed in the literature, it also should be noted that the excipients that are used in the present study are commercially available, which can lead to easier scale-up of the final formulations and reproducible physicochemical characterization [98]. We did not select natural polymers, i.e., chitosan or dextran. There are several challenges in the use of these natural polymers in terms of their stability, scalability, and immunogenicity [99]. However, direct comparisons with the present study’s results are not feasible due to variations in experimental conditions. Specifically, all previous studies used sheep and porcine nasal mucosa for ex vivo permeation experiments instead of rabbit nasal mucosa. Furthermore, the conduction of in vitro experiments differed in terms of RH loading doses, duration, and techniques used (method of dialysis bag).

Further studies are scheduled to evaluate the effect of dose and formulation on the in vitro release and ex vivo permeation of RH through rabbit nasal mucosa, as well as the in vivo serum and brain pharmacokinetic profiles of the developed formulations after nasal administration.

## 3. Materials and Methods

### 3.1. Materials

Pluronic^®^ F-127 (P407) was acquired from Sigma-Aldrich Chemical Co. (Merck, NJ, USA). Polysorbate 80 (Tw80), as well as the derivatives of β-CD (MβCD and HPβCD), were purchased from Sigma-Aldrich Chemical Co (St. Louis, MO, USA). All systems were prepared using HPLC-grade water (Fischer Scientific, Pittsburgh, PA, USA). Chloroform, pyrene, and other reagents used were of analytical grade and purchased from Fischer Scientific (Pittsburgh, PA, USA). Ropinirole hydrochloride was kindly donated by Uni-Pharma S.A (Athens, Greece). Pyrene was dissolved in the appropriate concentration (1 mM) in acetone. The molecular characteristics of the utilized components are presented in Table 7.

### 3.2. Methods

#### 3.2.1. Differential Scanning Calorimetry

DSC analyses were performed to determine the interactions between the molecules, i.e., between P407, Tw80 and CDs, in the solid state as a preformulative approach. The analyses were carried out using a DSC822^e^ Mettler-Toledo (Schwerzenbach, Switzerland) calorimeter calibrated with pure indium (T_m_ = 157 °C). Raw materials, including P407, Tw80, MβCD, and HPβCD, as well as their combinations (Tw80/MβCD at a 70:30 ratio, Tw80/HPβCD at a 70:30 ratio, P407/Tw80 at a 70:30 ratio, P407/MβCD at an 80:20 ratio, P407/HPβCD at an 80:20 ratio, (P407/Tw80)/MβCD and (P407/Tw80)/HPβCD at an 80:20 ratio) were examined. Each sample was analyzed by placing 2.0–6.0 mg of powder in a crucible, sealing it, and subjecting it to a constant temperature of 10 °C for equilibration. The samples were then heated at a rate of 10 °C/min from 10 °C to 300 °C under a steady flow of nitrogen. An empty aluminum crucible was used as a reference during the analyses. The data obtained from the DSC measurements (enthalpy changes ΔH_m/s_, characteristic transition temperatures T_onset,m/s_ and T_m/s_, and widths at half peak height of the C_p_ profiles ΔT_1/2,m/s_) were analyzed using Mettler-Toledo STAR^e^ software (version 9.20) and their R-squared values were determined.

#### 3.2.2. Preparation of Hybrid Systems

The colloidal dispersions of P407, P407/Tw80, P407/Tw80/MβCD, and P407/Tw80/HPβCD were prepared by the conventional thin-film hydration method. The polymer/surfactant weight ratio was 70:30 *w*/*w* and the further incorporation of CDs was achieved at a (polymer/surfactant)/CD weight ratio of 80:20 *w*/*w*. First, the appropriate amount of P407 was added to a dry round-bottom flask and was dissolved in the minimum volume of chloroform. A thin film formed inside the flask wall after the evaporation (under vacuum, 40 °C) of the organic solvent (Hei-VAP series CORE-heidolph^®^, Schwabach, Germany). Afterwards, the film was hydrated for half an hour using HPLC-grade water as a dispersion medium. Hydration was achieved by slow rotation of the flask in a water bath at 40 °C and the final concentration of the colloidal dispersion was equal to 10 mg/mL. The resultant samples were subjected to two 3 min sonication cycles (amplitude 70%, cycle 0.5 s), interrupted by a 3 min resting period and performed using a probe sonicator (Bandelin sonopuls, homogenizer, HD3200, Berlin, Germany). The preparation of binary P407/Tw80 and ternary P407/Tw80/CD systems was employed using the same protocol. Regarding binary systems, P407 and Tw80 were codissolved in chloroform at a molar ratio of 70:30 *w*/*w*. After the evaporation procedure, a thin film formed and was then hydrated with the appropriate volume of water. The preparation of ternary P407/Tw80/CD systems was conducted using the same protocol. P407 and Tw80 were dissolved in chloroform at the same molar ratio as in the binary system. The resulting mixture was subjected to evaporation, forming a thin film. CD solution in water was added at a molar ratio of (P407/Tw80)/CD equal to 80:20 *w*/*w*. The appropriate volume of water was added to reach a final colloidal concentration of 10 mg/mL.

#### 3.2.3. Physicochemical Characterization and Physical Stability Studies by Light Scattering Techniques

The average R_h_, the corresponding percentage of each population in each sample, and the z-potential of the colloidal dispersions obtained were investigated using DLS and ELS. Measurements were conducted using a wide-angle light scattering photometer by ALV GmbH, CGS-3. This setup consists of a He-Ne 22 mW laser source, a compact goniometer system with an Avalanche photodiode detector interfaced with an ALV/LSE-5003 electronics unit, and an ALV-5000/EPP multi-tau digital photon correlator. The results were analyzed using the Contin method. The measurements were implemented at a fixed scattering angle of 90° degrees and at a temperature of 25 °C after diluting 100 μL of each sample (10 mg/mL) with HPLC-grade water 30 times.

Following their preparation, the colloidal dispersions were stored in ambient glass vials at 4 °C. Their physicochemical characteristics were measured immediately after preparation (t = 0 days) and at selected times during a 30-day period under the above-mentioned storage conditions.

#### 3.2.4. Biological Stability of Hybrid Systems

The stealth properties of the prepared systems were also tested. A mixture of FBS and PBS at a weight ratio of 10:90 was used as a dispersion medium, simulating the physicochemical conditions of human plasma. Moreover, possible alterations in the particle size of hybrid systems in conditions that mimic the nasal cavity were also tested. For this reason, DLS experiments were conducted at 34 °C [89] using a buffer solution with a pH = 5.6 as a dispersion medium. For these studies, 100 μL of each system was diluted with 2900 μL of each dispersion medium.

#### 3.2.5. Fluorescence Spectroscopy

Fluorescence spectroscopy was employed to gather qualitative insights into the internal structure and microenvironment of the prepared systems in HPLC-grade water. Pyrene was used as a hydrophobic probe, capable of being incorporated into the hydrophobic domains of the hybrid platforms. A NanoLog Fluorometer (Horiba Jobin Yvon, Piscataway, NJ, USA) was used to record the pyrene emission spectra, with a laser diode serving as the excitation source (Nano LED, 440 nm, 100 ps pulse width) and a UV TBX-PMT series detector (250–850 nm) from Horiba Jobin Yvon (Piscataway, NJ, USA). A description of the utilized method is presented below.

Colloidal dispersions of P407, P407/Tw80, P407/Tw80/MβCD, and P407/Tw80/HPβCD were prepared at concentrations of 10 mg/mL. Following this, 3 μL of a pyrene stock solution (1 mM) was added to each colloidal dispersion. The dispersions were equilibrated for 24 h and the I_1_/I_3_ ratio was measured at each hybrid system concentration at temperatures of 25 °C, 37 °C, and 50 °C. Fluorescence spectra were collected in the range of 355–630 nm, with an excitation wavelength of 335 nm. Notably, no excimer formation was observed for the examined solutions.

#### 3.2.6. Microcalorimetry Analysis

Calorimetric analyses were performed using a microDSC III (Setaram, Lyon, France). After 20 min equilibration at 5 °C, the prepared systems (Section 3.2.2) underwent a heating ramp from 5 °C to 80 °C at 1 °C/min. Temperature (T_m_, °C) and enthalpy (ΔH, J/g of solution) were determined using the dedicated software of the instrument (Setsoft2000, Setaram). Specifically, the peak and the area of the transition were utilized for the respective calculations. All measurements were performed in triplicate.

#### 3.2.7. High-Resolution Ultrasound Spectroscopy

A HR-US 102 high-resolution spectrometer (Ultrasonic Scientific, Dublin, Ireland) was employed to measure the differential relative ultrasonic velocity of samples with respect to temperature at a frequency of 5.4 MHz, which was determined by a broad amplitude frequency scan. The ultrasonic cells were filled with approximately 2 mL of the prepared colloidal dispersions (Section 3.2.2) and a reference solution of water. Then, they were equilibrated at 5 °C for at least 20 min. The samples were then analyzed using the same thermal program utilized for mDSC analyses (i.e., from 5 °C to 80 °C at a rate of 1 °C/min), with temperature control provided by a HAAKE C25P thermostat (Thermo Electron Corporation, Langensebold, Hessen, Germany). Differential ultrasonic sound speed was calculated by subtracting the contribution of the reference signal from that of the sample cell. Sample transitions in ultrasonic sound speed were determined from the first derivative of the signal. All measurements were conducted in triplicate.

#### 3.2.8. Surface Tension Measurements

The colloidal systems (Section 3.2.2) underwent tensiometric analyses at a temperature of 20 °C using a DCA-100 contact angle tensiometer (First Ten Angstrom, Newark, CA, USA) according to the du Noüy ring method. Surface tension measurements were performed following the protocol outlined by Perinelli et al. [83]. All analyses were conducted in triplicate, and the results are presented as the mean ± SD of three independent measurements.

#### 3.2.9. Cryo-TEM Images

Cryo-TEM images were obtained using a Tecnai F20 X TWIN microscope (FEI Company, Hillsboro, OR, USA) equipped with a field emission gun and operating at an acceleration voltage of 200 kV. Images were recorded on a Gatan Rio 16 CMOS 4k camera (Gatan Inc., Pleasanton, CA, USA) and processed with Gatan Microscopy Suite (GMS) software (version 3.31.2360.0, Gatan Inc., Pleasanton, CA, USA). Specimen preparation was performed by vitrification of the aqueous solutions on grids with holey carbon film (Quantifoil R 2/2; Quantifoil Micro Tools GmbH, Großlöbichau, Germany). Prior to use, the grids were activated for 15 s in oxygen plasma using a Femto plasma cleaner (Diener Electronic, Ebhausen, Germany). Cryo-samples were prepared by applying a droplet (3 μL) of the suspension to the grid, blotting it with filter paper, and immediately freezing in liquid ethane using a fully automated Vitrobot Mark IV blotting device (Thermo Fisher Scientific, Waltham, MA, USA). After preparation, the vitrified specimens were kept under liquid nitrogen until they were inserted into a cryo-TEM-holder Gatan 626 (Gatan Inc., Pleasanton, CA, USA) and analyzed in the TEM at −178 °C.

#### 3.2.10. In Vitro Cytotoxicity

The biocompatibility and safety of the prepared systems were assessed by conducting in vitro cytotoxicity studies. The MTT assay was utilized to quantify the decrease in cell viability on HEK293 cell lines upon exposure to the prepared systems applying the protocol described in previous studies [72].

#### 3.2.11. Preformulation Studies, Preparation, and Physicochemical Characterization of RH Hybrid Systems

RH was used as a model drug to further study the loading and release properties of the prepared hybrid polymer/surfactant/CD systems. To optimize the amount of RH incorporated into the hybrid P407/Tw80/MβCD and P407/Tw80/HPβCD formulations, the alterations of the systems’ thermotropic behaviors using different weight ratios (P407/Tw80/CD)/RH (10:0.1, 10:0.5, 10:1, 10:5, and 10:10) were evaluated. For this reason, the systems were prepared by the dialysis method and after the evaporation of the solvent, DSC experiments took place, as described in Section 3.2.1. Following the selection of the most suitable system/RH weight ratios (10:1 and 10:5), the preparation of drug systems was achieved by the thin-film hydration method. For this reason, P407, Tw80, and an RH chloroform stock solution (the appropriate volume to achieve the weight ratios of 10:1 and 10:5) were added to a round-bottom flask. A thin film formed on the flask walls after the evaporation (under vacuum, 40 °C) of the organic solvent. MβCD or HPβCD solutions in water were added at a molar ratio of (P407/Tw80)/CD equal to 80:20 *w*/*w*. Water was added to achieve a final colloidal concentration of 10 mg/mL. The particle size of drug systems, using HPLC-grade water as a dispersion medium, was evaluated using the DLS technique as described in Section 3.2.3.

#### 3.2.12. Quantitative Analysis of RH

RH was quantified in the prepared systems and was determined in the samples of in vitro and ex vivo experiments using HPLC-PDA with a Shimadzu prominence system (Kyoto, Japan). The HPLC system included an LC-20AD Quaternary Gradient Pump with a degasser, an SIL-HT auto-sampler, and an SPD-M20A photo-diode array detector. Data acquisition and analysis were performed with LC solution^®^ software (LabSolutions, version 1.25 SP4, Kyoto, Japan). A reverse-phase Thermo C_18_ ODS Hypershil column (100 × 4.6 mm, 5 μm particle size) connected to a C_18_ precolumn (12.5 × 4.6 mm, 5 μm particle size) was used as the stationary phase. A 0.1 M potassium phosphate buffer, pH 2.5, was mixed with acetonitrile to prepare the mobile phase, consisting of a 60:40 *v/v* proportion of acetonitrile/potassium phosphate buffer. The flow rate was set to 0.8 mL/min. The injection volume was 30 μL and λ_max_ was set at 245 nm. The calibration curve was established using RH’s methanolic stock solution (0.2 mg/mL) and the mobile phase (acetonitrile/potassium phosphate buffer 60:40 *v*/*v*) for all dilutions. The range of calibration curve samples was 0.5 to 10 μg/mL for RH and 0.1 to 2 μg/mL for the analysis of samples obtained from in vitro and ex vivo experiments, respectively.

#### 3.2.13. RH’s Release from the Hybrid Formulations by In Vitro Diffusion Experiments

The incorporation of RH into the tested ternary systems (P407/Tw80/MβCD and P407/Tw80/HPβCD) was achieved at two different concentrations (1 mg/mL and 5 mg/mL) based on the results of DSC experiments in which the concentrations of the drug in the systems varied. In vitro diffusion experiments were carried out using regenerated cellulose membranes with a molecular mass cut-off of 1000 Da and Franz-type diffusion cells (Crown Glass, Somerville, MA, USA). The protocol for the preparation of the membranes included their immersion in distilled water for 15 min, followed by its replacement with fresh distilled water and soaking for an additional 30 min. Then, the membranes were transferred to a beaker with PBS (pH 7.4) and soaked for 15 min. After this pretreatment, the membranes were cut into squares of 1 cm^2^ surface in order to completely cover the Franz cells’ diffusion area (0.636 cm^2^). The Franz cells were assembled by filling the receptor compartment with 5 mL of PBS and the membrane was mounted between the receptor and donor compartments. A magnetic stirrer was added to the receptor, and the two parts were kept together with a metal clamp. The assembled system was allowed to equilibrate at 34 °C for 15 min, which corresponds to the temperature of the nasal cavity. 

The dose of the colloidal dispersions examined was equal to 0.05 mg. More specifically, 50 μL of the formulation with a concentration of API equal to 1 mg/mL (corresponding to a weight ratio of (P407/Tw80/CD)/RH of 10:1 *w*/*w*) was placed in the donor compartment and diluted with 50 μL of PBS (pH 5.6). For formulation with an API concentration of 5 mg/mL (corresponding to a weight ratio of (P407/Tw80/CD)/RH of 10:5 *w*/*w*), 10 μL of the formulation was placed in the donor compartment and diluted with 90 μL of PBS (pH 5.6).

An RH solution with a concentration of 0.5 mg/mL in buffer with pH 5.6 was also prepared and tested by introducing 100 μL of the solution into the donor compartment. The donor and receptor compartments were both covered with Parafilm^®^ to prevent evaporation. All experiments lasted 4 h. At specific time intervals (15, 30, 45, 60, 90, 120, 150, 180, 210, and 240 min), 0.5 mL was sampled from the receptor compartment and replaced by an equal volume of fresh PBS (pH 7.4). At the end of the experiment, the residual formulation in the donor compartment was quantitatively collected and diluted to determine the remaining RH and calculate the mass balance. The cellulose membranes were washed with an H_2_O/methanol (50:50) solution to retrieve the amount of RH remaining in the membrane and the extract. All samples were analyzed by HPLC-PDA (Section 3.2.13).

The diffusion area (*A*) of the Franz cell is equal to 0.636 cm^2^. *J* across the artificial membrane from the donor to the receptor compartment was calculated using the slopes obtained by regression analysis of the amount of RH (*Q*) permeated per unit area (*A*) vs. time, according to Equation (1) [100].

(1) $$J=\frac{dQ}{dt\times A}$$

#### 3.2.14. RH’s Ex Vivo Mucosal Permeation from the Hybrid Formulations

Ex vivo mucosal permeation experiments were performed using Franz diffusion cells and rabbit nasal mucosa as a model barrier [101]. Nasal mucosa was extracted on the day of the experiment from rabbit heads collected from a local slaughterhouse (Athens, Greece). Mucosa extraction was carried out according to Papakyriakopoulou et al. [3] and the barrier consisted of both the epithelial barrier and connective tissue. In addition, the values of the flux, *J*, across the nasal mucosa barrier to the receptor compartment of the Franz cells were obtained with Equation (2). *P_app_* was calculated by dividing *J* by the initial drug concentration in the donor compartment of the Franz cells (*C*_0_), as described by Equation (2) [100]:

(2) $$P_{app}=\frac{J}{C_{0}}$$

#### 3.2.15. Statistical Analysis

The experimental data were assessed using the GraphPad Prism 5.0 software package (GraphPad Software, version 5.01). A significance level of *p* < 0.05 was chosen, and all tests were two-tailed with a 95% CI. To identify outliers, the interquartile range (IQR) method with a 1.5 × IQR threshold was employed, but no outliers were detected. One-way ANOVA with Bonferroni post hoc tests for multiple comparisons was conducted to identify potential statistically significant variations among all the pairs of compared groups. The results are presented as mean ± SD for the results of in vitro cytotoxicity studies and in vitro diffusion experiments and mean ± SEM for ex vivo permeation experiments. Permeation values were subjected to statistical comparisons both among the tested formulations and at each time point within each formulation.

## 4. Conclusions

This study represents the first utilization of hybrid nanoparticles consisting of commercially available polymers, surfactants, and CDs for drug delivery and targeting of RH. Differential scanning calorimetry experiments revealed a wide range of interactions between the compounds, which were confirmed by the application of mDSC analysis and HR-US. The results obtained from fluorescence spectroscopy confirm that P407 is a thermoresponsive polymer whose properties are influenced by temperature. Concerning the surface tension of the systems, the presence of the surfactant and β-CD derivatives did not cause a significant alteration to the surface tension values of P407 hybrid systems. The structures of the systems, as visualized in cryo-TEM images, exhibited a spherical configuration. In vitro cytotoxicity evaluation revealed that the prepared systems did not exhibit any cytotoxicity at low concentrations. However, as the dose was increased, a correlation between dose and cytotoxicity was observed on HEK293 cell lines, with the degree of cytotoxicity varying based on the materials’ composition. The incorporation of RH into the hybrid systems was achieved in two different concentrations (1 mg/mL and 5 mg/mL), based on the results of DSC. In vitro diffusion experiments revealed that more than 90% of the loading dose was released. Mass balance was found to be higher than 90%, indicating the stability of RH under conditions simulating the nasal cavity. Ex vivo permeation experiments through rabbit nasal mucosa revealed the superiority of the prepared hybrid systems in comparison to the RH solution. These encouraging in vitro and ex vivo results indicate the potential of these nanoparticle formulations for drug delivery applications. Specifically, these hybrid systems, which combine a polymer, surfactant, and CDs, present appealing opportunities for the nose-to-brain delivery of RH in PAD, as revealed by the results of the present study. Further studies are ongoing to evaluate the effect of dosage on the in vitro release and ex vivo permeation of the developed formulations through rabbit nasal mucosa, as well as their in vivo serum and brain pharmacokinetic profiles after nasal administration.

## Figures and Tables

**Table 1:** The physicochemical properties of the prepared systems on the day of their preparation.

Colloidal Dispersions	*w*/*w*	R_h (Cumulant)_ (nm) ^1^	PDI ^2^	Number of Peaks	R_h (Contin)_ (nm) ^3^	Weight of Peak (%)	z-Potential (mV)
P407	-	97	0.4_9_	3	(1) 4 (2) 39 (3) 598	(1) 6% (2) 38% (3) 55%	−20.5 ± 6.0
P407/Tw80	70:30	18	0.5_2_	1	29	100%	−6.1 ± 2.0
(P407/Tw80)/MβCD	80:20	106	0.3_2_	2	(1) 8 (2) 104	(1) 3% (2) 97%	−12.9 ± 12.0
(P407/Tw80)/HPβCD	80:20	100	0.3_0_	2	(1) 9 (2) 114	(1) 3% (2) 97%	−6.9 ± 8.4

^1^ R_h_ indicates the average hydrodynamic radius of three replicates of each sample measured by the Cumulant method; ^2^ PDI indicates the average polydispersity index, and the first decimal number is the significant one; ^3^ R_h_ indicates the average hydrodynamic radius of three replicates of each sample measured by the Contin method.

**Table 2:** I_1_/I_3_ values of systems analyzed at different temperatures (25 °C, 37 °C, 50 °C).

System	*w*/*w*	Temperature	I_1_/I_3_
P407	-	25 °C	1.28
P407	-	37 °C	1.30
P407	-	50 °C	1.19
P407/Tw80	70:30	25 °C	1.19
P407/Tw80	70:30	37 °C	1.31
P407/Tw80	70:30	50 °C	1.24
(P407/Tw80)/MβCD	80:20	25 °C	1.29
(P407/Tw80)/MβCD	80:20	37 °C	1.35
(P407/Tw80)/MβCD	80:20	50 °C	1.20
(P407/Tw80)/HPβCD	80:20	25 °C	1.24
(P407/Tw80)/HPβCD	80:20	37 °C	1.37
(P407/Tw80)/HPβCD	80:20	50 °C	1.41

**Table 3:** Thermodynamic parameters (transition temperature, °C, and associated enthalpy, J/g, of solution) and surface tension (mN/m) for the prepared formulations as calculated from mDSC, HR-US, and surface tension measurements.

Sample	mDSC		HR-US (Sound Speed)	Surface Tension Measurements
	Temperature (°C)	Enthalpy (J/g)	Temperature (°C)	Surface Tension (mN/m)
P407	28.05 ± 0.11	0.175 ± 0.007	30.07 ± 1.11	38.61 ± 0.28
P407/Tw80	24.35 ± 0.36	0.220 ± 0.016	27.92 ± 1.34	37.41 ± 0.31
P407/Tw80/MβCD	27.06 ± 0.23	0.116 ± 0.009	27.18 ± 1.43	38.26 ± 0.28
P407/Tw80/HPβCD	25.53 ± 0.16	0.101 ± 0.008	27.57 ± 1.36	37.23 ± 0.87

**Table 4:** Physicochemical characteristics of RH systems measured on the day of their preparation.

Colloidal Dispersions	*w*/*w*	R_h (Cumulant)_ (nm) ^1^	PDI ^2^	Number of Peaks	R_h (Contin)_ (nm) ^3^	Weight of Peak (%)
(P407/Tw80/MβCD)/RH	10:1	80	0.5_1_	2	(1) 4 (2) 99	(1) 7% (2) 92%
(P407/Tw80/MβCD)/RH	10:5	68	0.4_8_	2	(1) 8 (2) 211	(1) 20% (2) 80%
(P407/Tw80/HPβCD)/RH	10:1	53	0.5_1_	2	(1) 6 (2) 60	(1) 12% (2) 88%
(P407/Tw80/HPβCD)/RH	10:5	29	0.5_3_	2	(1) 6 (2) 61	(1) 20% (2) 80%

^1^ R_h_ indicates the average hydrodynamic radius of three replicates of each sample measured by the Cumulant method; ^2^ PDI indicates the average polydispersity index, and the first decimal number is the significant one; ^3^ R_h_ indicates the average hydrodynamic radius of three replicates of each sample measured by the Contin method.

**Table 5:** Percentage (%) of RH loading dose permeated for the tested formulations (mean ± SD, *n* = 3); mass balance (%) of RH in each formulation (mean ± SD, *n* = 3); and membrane retention data of each formulation expressed as the percentage (%) of the loading dose retained by the cellulose membrane (mean ± SD, *n* = 3).

System		*w*/*w*	Permeated RH (% Loading Dose)	Mass Balance (%)	% of RH Dose Retained by Cellulose Membrane
F1	(P407/Tw80/MβCD)/RH	10:1	93.56 ± 0.19	96.45 ± 0.85	0.50 ± 0.07
F2	(P407/Tw80/MβCD)/RH	10:5	92.09 ± 0.76	95.90 ± 2.52	0.40 ± 0.13
F3	(P407/Tw80/HPβCD)/RH	10:1	90.47 ± 4.46	93.60 ± 4.99	0.51 ± 0.01
F4	(P407/Tw80/HPβCD)/RH	10:5	95.81 ± 1.48	99.97 ± 1.43	0.37 ± 0.02
Control	RH solution (0.5 mg/mL)	-	96.93 ± 2.11	99.52 ± 2.33	0.37 ± 0.04

**Table 6:** Percentage (%) of RH loading dose permeated for the tested formulations (mean ± SEM, *n* = 4); mass balance of RH in each formulation (mean ± SEM, *n* = 4); and membrane retention data of each formulation expressed as the percentage (%) of the loading dose retained by the nasal mucosa barrier (mean ± SEM, *n* = 4).

System		Weight Ratio	Permeated RH (% Loading Dose)	Mass Balance (%)	% of RH Dose Retained by Nasal Mucosa Barrier
F1	(P407/Tw80/MβCD)/RH	10:1	29.24 ± 1.60	87.85 ± 0.61	13.47 ± 0.86
F2	(P407/Tw80/MβCD)/RH	10:5	28.58 ± 1.03	96.29 ± 3.69	14.73 ± 2.02
F3	(P407/Tw80/HPβCD)/RH	10:1	22.26 ± 3.37	86.82 ± 1.24	25.80 ± 3.79
F4	(P407/Tw80/HPβCD)/RH	10:5	28.26 ± 0.76	90.53 ± 2.03	15.76 ± 1.80
Control	RH solution (0.5 mg/mL)	-	19.62 ± 0.53	81.78 ± 2.36	18.10 ± 0.34

**Table 7:** Molecular characteristics of P407, Tween 80, MβCD, HPβCD, and RH.

	P407	Tw80	MβCD	HPβCD	RH
Molecular formula	C_572_H_1146_O_259_	C_64_H_124_O_26_	C_54_H_94_O_35_	C_63_H_112_O_42_	C_16_H_25_ClN_2_O
MW (g/mol)	12600	1310	1303.3	1541.54	296.83

## Data Availability

Data are contained within the article and Supplementary Materials.

## Supplementary Materials

The following supporting information can be downloaded at: https://www.mdpi.com/article/10.3390/ijms25021162/s1.

## Abbreviations

APIs	active pharmaceutical ingredients
BBB	blood–brain barrier
CDs	cyclodextrins
CI	confidence interval
DLS	dynamic light scattering
DSC	differential scanning calorimetry
ELS	electrophoretic light scattering
FBS	fetal bovine serum
FTIR	Fourier-transform infrared spectroscopy
GI	gastrointestinal
HPLC	high-performance liquid chromatography
HPβCD	hydroxy-propyl-β-CD
HR-US	high-resolution ultrasound spectroscopy
IQR	ιnterquartile range
J	flux
ΜβCD	methyl-β-CD
mDSC	microcalorimetry
PAD	Parkinson’s disease
*P_app_*	apparent permeability
PBS	phosphate buffer solution
P407	poloxamer 407
PDI	polydispersity index
PEO	polyethylene oxide
PPO	poly(propylene oxide)
RH	ropinirole hydrochloride
R_h_	hydrodynamic radius
SD	standard deviation
SEM	standard error of the mean
T_m_	main transition temperature
Tw80	Tween 80
z-potential	zeta potential
ΔH	enthalpy
ΔΤ_1/2_	half width at half peak height of the transition

## References

[1] Schneider R.B., Iourinets J., Richard I.H. (2017). Parkinson’s disease psychosis: Presentation, diagnosis and management. Neurodegener. Dis. Manag..

[2] Kumar R., Aadil K.R., Mondal K., Mishra Y.K., Oupicky D., Ramakrishna S., Kaushik A. (2022). Neurodegenerative disorders management: State-of-art and prospects of nano-biotechnology. Crit. Rev. Biotechnol..

[3] Papakyriakopoulou P., Manta K., Kostantini C., Kikionis S., Banella S., Ioannou E., Christodoulou E., Rekkas D.M., Dallas P., Vertzoni M. (2021). Nasal powders of quercetin-β-cyclodextrin derivatives complexes with mannitol/lecithin microparticles for Nose-to-Brain delivery: In vitro and ex vivo evaluation. Int. J. Pharm..

[4] Di Prima G., Campisi G., De Caro V. (2020). Amorphous Ropinirole-Loaded Mucoadhesive Buccal Film: A Potential Patient-Friendly Tool to Improve Drug Pharmacokinetic Profile and Effectiveness. J. Pers. Med..

[5] Lai K.L., Fang Y., Han H., Li Q., Zhang S., Li H.Y., Chow S.F., Lam T.N., Lee W.Y.T. (2018). Orally-dissolving film for sublingual and buccal delivery of ropinirole. Colloids Surf. B Biointerfaces.

[6] Kontogiannidou E., Andreadis D.A., Zografos A.L., Nazar H., Klepetsanis P., van der Merwe S.M., Fatouros D.G. (2017). Ex vivo buccal drug delivery of ropinirole hydrochloride in the presence of permeation enhancers: The effect of charge. Pharm. Dev. Technol..

[7] De Caro V., Giandalia G., Siragusa M.G., Sutera F.M., Giannola L.I. (2012). New prospective in treatment of Parkinson’s disease: Studies on permeation of ropinirole through buccal mucosa. Int. J. Pharm..

[8] Hua S. (2019). Advances in Nanoparticulate Drug Delivery Approaches for Sublingual and Buccal Administration. Front. Pharmacol..

[9] Macedo A.S., Castro P.M., Roque L., Thomé N.G., Reis C.P., Pintado M.E., Fonte P. (2020). Novel and revisited approaches in nanoparticle systems for buccal drug delivery. J. Control. Release.

[10] Kulkarni A.D., Vanjari Y.H., Sancheti K.H., Belgamwar V.S., Surana S.J., Pardeshi C.V. (2015). Nanotechnology-mediated nose to brain drug delivery for Parkinson’s disease: A mini review. J. Drug Target..

[11] Rao M., Agrawal D.K., Shirsath C. (2017). Thermoreversible mucoadhesive in situ nasal gel for treatment of Parkinson’s disease. Drug Dev. Ind. Pharm..

[12] Imran M., Almehmadi M., Alsaiari A.A., Kamal M., Alshammari M.K., Alzahrani M.O., Almaysari F.K., Alzahrani A.O., Elkerdasy A.F., Singh S.K. (2023). Intranasal Delivery of a Silymarin Loaded Microemulsion for the Effective Treatment of Parkinson’s Disease in Rats: Formulation, Optimization, Characterization, and In Vivo Evaluation. Pharmaceutics.

[13] Lin H., Xie L., Lv L., Chen J., Feng F., Liu W., Han L., Liu F. (2023). Intranasally administered thermosensitive gel for brain-targeted delivery of rhynchophylline to treat Parkinson’s disease. Colloids Surf. B Biointerfaces.

[14] Kim K.H. (2023). Intranasal delivery of mitochondrial protein humanin rescues cell death and promotes mitochondrial function in Parkinson’s disease. Theranostics.

[15] Sridhar V., Wairkar S., Gaud R., Bajaj A., Meshram P. (2018). Brain targeted delivery of mucoadhesive thermosensitive nasal gel of selegiline hydrochloride for treatment of Parkinson’s disease. J. Drug Target..

[16] Chroni A., Mavromoustakos T., Pispas S. (2020). Biocompatible PEO-b-PCL Nanosized Micelles as Drug Carriers: Structure and Drug-Polymer Interactions. Nanomaterials.

[17] Liechty W.B., Kryscio D.R., Slaughter B.V., Peppas N.A. (2010). Polymers for drug delivery systems. Annu. Rev. Chem. Biomol. Eng..

[18] Wang H., Williams G.R., Wu J., Wu J., Niu S., Xie X., Li S., Zhu L.M. (2019). Pluronic F127-based micelles for tumor-targeted bufalin delivery. Int. J. Pharm..

[19] Merkus F.W., Verhoef J.C., Marttin E., Romeijn S.G., van der Kuy P.H., Hermens W.A., Schipper N.G. (1999). Cyclodextrins in nasal drug delivery. Adv. Drug Deliv. Rev..

[20] Khodaverdi E., Heidari Z., Tabassi S.A., Tafaghodi M., Alibolandi M., Tekie F.S., Khameneh B., Hadizadeh F. (2015). Injectable supramolecular hydrogel from insulin-loaded triblock PCL-PEG-PCL copolymer and γ-cyclodextrin with sustained-release property. AAPS PharmSciTech.

[21] Xia Y., Li L., Huang X., Wang Z., Zhang H., Gao J., Du Y., Chen W., Zheng A. (2020). Performance and toxicity of different absorption enhancers used in the preparation of Poloxamer thermosensitive in situ gels for ketamine nasal administration. Drug Dev. Ind. Pharm..

[22] Manta K., Papakyriakopoulou P., Nikolidaki A., Balafas E., Kostomitsopoulos N., Banella S., Colombo G., Valsami G. (2023). Comparative Serum and Brain Pharmacokinetics of Quercetin after Oral and Nasal Administration to Rats as Lyophilized Complexes with β-Cyclodextrin Derivatives and Their Blends with Mannitol/Lecithin Microparticles. Pharmaceutics.

[23] Papakyriakopoulou P., Balafas E., Colombo G., Rekkas D.M., Kostomitsopoulos N., Valsami G. (2023). Nose-to-Brain delivery of donepezil hydrochloride following administration of an HPMC-Me-β-CD-PEG400 nasal film in mice. J. Drug Deliv. Sci. Technol..

[24] Lewis A.L., Jordan F., Illum L. (2013). CriticalSorb™: Enabling systemic delivery of macromolecules via the nasal route. Drug Deliv. Transl. Res..

[25] Valero M., Castiglione F., Mele A., da Silva M.A., Grillo I., González-Gaitano G., Dreiss C.A. (2016). Competitive and Synergistic Interactions between Polymer Micelles, Drugs, and Cyclodextrins: The Importance of Drug Solubilization Locus. Langmuir.

[26] Haley R.M., Gottardi R., Langer R., Mitchell M.J. (2020). Cyclodextrins in drug delivery: Applications in gene and combination therapy. Drug Deliv. Transl. Res..

[27] Kerwin B.A. (2008). Polysorbates 20 and 80 used in the formulation of protein biotherapeutics: Structure and degradation pathways. J. Pharm. Sci..

[28] Cirpanli Y., Bilensoy E., Lale Doğan A., Caliş S. (2009). Comparative evaluation of polymeric and amphiphilic cyclodextrin nanoparticles for effective camptothecin delivery. Eur. J. Pharm. Biopharm..

[29] Kuplennik N., Sosnik A. (2019). Enhanced Nanoencapsulation of Sepiapterin within PEG-PCL Nanoparticles by Complexation with Triacetyl-Beta Cyclodextrin. Molecules.

[30] Li X., Yu Y., Ji Q., Qiu L. (2015). Targeted delivery of anticancer drugs by aptamer AS1411 mediated Pluronic F127/cyclodextrin-linked polymer composite micelles. Nanomedicine.

[31] Gao Y., Li G., Zhou Z., Guo L., Liu X. (2017). Supramolecular assembly of poly(β-cyclodextrin) block copolymer and benzimidazole-poly(ε-caprolactone) based on host-guest recognition for drug delivery. Colloids Surf. B Biointerfaces.

[32] Cui X., Wang N., Wang H., Li G., Tao Q. (2019). pH sensitive supramolecular vesicles from cyclodextrin graft copolymer and benzimidazole ended block copolymer as dual drug carriers. Int. J. Polym. Mater. Polym. Biomater..

[33] Zhang L., Lu J., Jin Y., Qiu L. (2014). Folate-conjugated beta-cyclodextrin-based polymeric micelles with enhanced doxorubicin antitumor efficacy. Colloids Surf. B Biointerfaces.

[34] Poudel A.J., He F., Huang L., Xiao L., Yang G. (2018). Supramolecular hydrogels based on poly (ethylene glycol)-poly (lactic acid) block copolymer micelles and α-cyclodextrin for potential injectable drug delivery system. Carbohydr. Polym..

[35] Li X., Liu H., Li J., Deng Z., Li L., Liu J., Yuan J., Gao P., Yang Y., Zhong S. (2019). Micelles via self-assembly of amphiphilic beta-cyclodextrin block copolymers as drug carrier for cancer therapy. Colloids Surf. B Biointerfaces.

[36] Simões S.M., Veiga F., Torres-Labandeira J.J., Ribeiro A.C., Sandez-Macho M.I., Concheiro A., Alvarez-Lorenzo C. (2012). Syringeable Pluronic-α-cyclodextrin supramolecular gels for sustained delivery of vancomycin. Eur. J. Pharm. Biopharm..

[37] Vasconcelos T., Prezotti F., Araújo F., Lopes C., Loureiro A., Marques S., Sarmento B. (2021). Third-generation solid dispersion combining Soluplus and poloxamer 407 enhances the oral bioavailability of resveratrol. Int. J. Pharm..

[38] Prasad Kushwaha J., Baidya D., Patil S. (2019). Harmine-loaded galactosylated pluronic F68-gelucire 44/14 mixed micelles for liver targeting. Drug Dev. Ind. Pharm..

[39] Patil S., Choudhary B., Rathore A., Roy K., Mahadik K. (2015). Enhanced oral bioavailability and anticancer activity of novel curcumin loaded mixed micelles in human lung cancer cells. Phytomedicine.

[40] Kanade R., Boche M., Pokharkar V. (2018). Self-Assembling Raloxifene Loaded Mixed Micelles: Formulation Optimization, In Vitro Cytotoxicity and In Vivo Pharmacokinetics. AAPS PharmSciTech.

[41] Hou J., Wang J., Sun E., Yang L., Yan H.M., Jia X.B., Zhang Z.H. (2016). Preparation and evaluation of icariside II-loaded binary mixed micelles using Solutol HS15 and Pluronic F127 as carriers. Drug Deliv..

[42] Barcia E., Boeva L., García-García L., Slowing K., Fernández-Carballido A., Casanova Y., Negro S. (2017). Nanotechnology-based drug delivery of ropinirole for Parkinson’s disease. Drug Deliv..

[43] Dudhipala N., Gorre T. (2020). Neuroprotective Effect of Ropinirole Lipid Nanoparticles Enriched Hydrogel for Parkinson’s Disease: In Vitro, Ex Vivo, Pharmacokinetic and Pharmacodynamic Evaluation. Pharmaceutics.

[44] Chiu M.H., Prenner E.J. (2011). Differential scanning calorimetry: An invaluable tool for a detailed thermodynamic characterization of macromolecules and their interactions. J. Pharm. Bioallied Sci..

[45] Knopp M.M., Löbmann K., Elder D.P., Rades T., Holm R. (2016). Recent advances and potential applications of modulated differential scanning calorimetry (mDSC) in drug development. Eur. J. Pharm. Sci. Off. J. Eur. Fed. Pharm. Sci..

[46] Pérez-González M.L.L., González-de la Rosa C.H., Pérez-Hernández G., Beltrán H.I. (2020). Nanostructured oleic acid/polysorbate 80 emulsions with diminished toxicity in NL-20 cell line: Insights of potential drug carriers. Colloids Surf. B Biointerfaces.

[47] Al-Sabagh A.M., Nasser N.M., Migahed M.A., Kandil N.G. (2011). Effect of chemical structure on the cloud point of some new non-ionic surfactants based on bisphenol in relation to their surface active properties. Egypt. J. Pet..

[48] De Melo C.M., Da Silva A.L., De Melo K.R., Da Silva P.C.D., de Souza M.L., de Sousa A.L.M.D., Rabello M.M., Véras L.M.C., Rolim L.A., Rolim Neto P.J. (2021). In silico and in vitro study of epiisopiloturine/hydroxypropyl-β-cyclodextrin inclusion complexes obtained by different methods. J. Drug Deliv. Sci. Technol..

[49] Palli V., Leonis G., Zoupanou N., Georgiou N., Chountoulesi M., Naziris N., Tzeli D., Demetzos C., Valsami G., Marousis K.D. (2022). Losartan Interactions with 2-Hydroxypropyl-β-CD. Molecules.

[50] Figueiras A., Cardoso O., Veiga F., Carvalho R., Ballarò G. (2015). Pharmaceutica Analytica Acta Preparation and characterization of Trimethoprim inclusion complex with Methyl-β-Cyclodextrin and determination of its antimicrobial activity. Pharm. Anal. Acta.

[51] Danciu C., Soica C., Csanyi E., Ambrus R., Feflea S., Peev C., Dehelean C. (2012). Changes in the anti-inflammatory activity of soy isoflavonoid genistein versus genistein incorporated in two types of cyclodextrin derivatives. Chem. Cent. J..

[52] Ribeiro A., Figueiras A., Santos D., Veiga F. (2008). Preparation and solid-state characterization of inclusion complexes formed between miconazole and methyl-beta-cyclodextrin. AAPS PharmSciTech.

[53] Soares da Silva L.F., do Carmo F.A., de Almeida Borges V.R., Monteiro L.M., Rodrigues C.R., Cabral L.M., de Sousa V.P. (2011). Preparation and evaluation of lidocaine hydrochloride in cyclodextrin inclusion complexes for development of stable gel in association with chlorhexidine gluconate for urogenital use. Int. J. Nanomed..

[54] Talegaonkar S., Khan A., Khar R., Ahmad F., Iqbal Z. (2007). Development and characterization of paracetamol complexes with Hydroxypropyl-β-Cyclodextrin. Iran. J. Pharm. Res..

[55] Alghaith A.F., Mahrous G.M., Zidan D.E., Alhakamy N.A., Alamoudi A.J., Radwan A.A. (2021). Preparation, characterization, dissolution, and permeation of flibanserin—2-HP-β-cyclodextrin inclusion complexes. Saudi Pharm. J..

[56] Gavini E., Spada G., Rassu G., Cerri G., Brundu A., Cossu M., Sorrenti M., Giunchedi P. (2011). Development of solid nanoparticles based on hydroxypropyl-β-cyclodextrin aimed for the colonic transmucosal delivery of diclofenac sodium. J. Pharm. Pharmacol..

[57] Geng Q., Li T., Wang X., Chu W., Cai M., Xie J., Ni H. (2019). The mechanism of bensulfuron-methyl complexation with β-cyclodextrin and 2-hydroxypropyl-β-cyclodextrin and effect on soil adsorption and bio-activity. Sci. Rep..

[58] Mazyed E.A., Badria F.A., ElNaggar M.H., El-Masry S.M., Helmy S.A. (2022). Development of Cyclodextrin-Functionalized Transethoniosomes of 6-Gingerol: Statistical Optimization, In Vitro Characterization and Assessment of Cytotoxic and Anti-Inflammatory Effects. Pharmaceutics.

[59] Dubey P., Barker S.A., Craig D.Q.M. (2020). Design and Characterization of Cyclosporine A-Loaded Nanofibers for Enhanced Drug Dissolution. ACS Omega.

[60] Palacio J., Agudelo N., Lopez B. (2016). PLA/Pluronic (R) nanoparticles as potential oral delivery systems: Preparation, colloidal and chemical stability, and loading capacity. J. Appl. Polym. Sci..

[61] Saher O., Ghorab D.M., Mursi N.M. (2016). Preparation and in vitro/in vivo evaluation of antimicrobial ocular in situ gels containing a disappearing preservative for topical treatment of bacterial conjunctivitis. Pharm. Dev. Technol..

[62] da Rocha M.C.O., da Silva P.B., Radicchi M.A., Andrade B.Y.G., de Oliveira J.V., Venus T., Merker C., Estrela-Lopis I., Longo J.P.F., Báo S.N. (2020). Docetaxel-loaded solid lipid nanoparticles prevent tumor growth and lung metastasis of 4T1 murine mammary carcinoma cells. J. Nanobiotechnol..

[63] Sayed S., Elsharkawy F.M., Amin M.M., Shamsel-Din H.A., Ibrahim A.B. (2021). Brain targeting efficiency of intranasal clozapine-loaded mixed micelles following radio labeling with Technetium-99m. Drug Deliv..

[64] Pawar S., Pande V. (2015). Oleic Acid Coated Gelatin Nanoparticles Impregnated Gel for Sustained Delivery of Zaltoprofen: Formulation and Textural Characterization. Adv. Pharm. Bull..

[65] Lazaratos M., Karathanou K., Mainas E., Chatzigoulas A., Pippa N., Demetzos C., Cournia Z. (2020). Coating of magnetic nanoparticles affects their interactions with model cell membranes. Biochim. Biophys. Acta Gen. Subj..

[66] Zhang J., Ma P.X. (2009). Polymeric core-shell assemblies mediated by host-guest interactions: Versatile nanocarriers for drug delivery. Angew. Chem. Int. Ed..

[67] Santos Akkari A.C., Ramos Campos E.V., Keppler A.F., Fraceto L.F., de Paula E., Tófoli G.R., de Araujo D.R. (2016). Budesonide-hydroxypropyl-β-cyclodextrin inclusion complex in binary poloxamer 407/403 system for ulcerative colitis treatment: A physico-chemical study from micelles to hydrogels. Colloids Surf. B Biointerfaces.

[68] Lin H.R., Chang P.C. (2013). Novel pluronic-chitosan micelle as an ocular delivery system. J. Biomed. Mater. Res. B Appl. Biomater..

[69] Bin Jardan Y.A., Ahad A., Raish M., Al-Mohizea A.M., Al-Jenoobi F.I. (2023). Microwave-Assisted Formation of Ternary Inclusion Complex of Pterostilbene. Pharmaceuticals.

[70] Di Donato C., Iacovino R., Isernia C., Malgieri G., Varela-Garcia A., Concheiro A., Alvarez-Lorenzo C. (2020). Polypseudorotaxanes of Pluronic^®^ F127 with Combinations of α- and β-Cyclodextrins for Topical Formulation of Acyclovir. Nanomaterials.

[71] Kalogeropoulou F., Papailiou D., Protopapa C., Siamidi A., Tziveleka L.A., Pippa N., Vlachou M. (2023). Design and Development of Low- and Medium-Viscosity Alginate Beads Loaded with Pluronic(^®^) F-127 Nanomicelles. Materials.

[72] Kontogiannis O., Selianitis D., Perinelli D.R., Bonacucina G., Pippa N., Gazouli M., Pispas S. (2022). Non-Ionic Surfactant Effects on Innate Pluronic 188 Behavior: Interactions, and Physicochemical and Biocompatibility Studies. Int. J. Mol. Sci..

[73] Li F., Wen Y., Zhang Y., Zheng K., Ban J., Xie Q., Wen Y., Liu Q., Chen F., Mo Z. (2019). Characterisation of 2-HP-β-cyclodextrin-PLGA nanoparticle complexes for potential use as ocular drug delivery vehicles. Artif. Cells Nanomed. Biotechnol..

[74] Chountoulesi M., Pippa N., Pispas S., Chrysina E.D., Forys A., Trzebicka B., Demetzos C. (2018). Cubic lyotropic liquid crystals as drug delivery carriers: Physicochemical and morphological studies. Int. J. Pharm..

[75] Li W., Chountoulesi M., Antoniadi L., Angelis A., Lei J., Halabalaki M., Demetzos C., Mitakou S., Skaltsounis L.A., Wang C. (2022). Development and physicochemical characterization of nanoliposomes with incorporated oleocanthal, oleacein, oleuropein and hydroxytyrosol. Food Chem..

[76] Bonechi C., Donati A., Tamasi G., Pardini A., Rostom H., Leone G., Lamponi S., Consumi M., Magnani A., Rossi C. (2019). Chemical characterization of liposomes containing nutraceutical compounds: Tyrosol, hydroxytyrosol and oleuropein. Biophys. Chem..

[77] Wong C.Y., Martinez J., Zhao J., Al-Salami H., Dass C.R. (2020). Development of orally administered insulin-loaded polymeric-oligonucleotide nanoparticles: Statistical optimization and physicochemical characterization. Drug Dev. Ind. Pharm..

[78] Akbar S., Anwar A., Ayish A., Elliott J.M., Squires A.M. (2017). Phytantriol based smart nano-carriers for drug delivery applications. Eur. J. Pharm. Sci..

[79] Pippa N., Skouras A., Naziris N., Biondo F., Tiboni M., Katifelis H., Gazouli M., Demetzos C., Casettari L. (2020). Incorporation of PEGylated δ-decalactone into lipid bilayers: Thermodynamic study and chimeric liposomes development. J. Liposome Res..

[80] Selianitis D., Pispas S. (2021). Multi-responsive poly(oligo(ethylene glycol)methyl methacrylate)-co-poly(2-(diisopropylamino)ethyl methacrylate) hyperbranched copolymers via reversible addition fragmentation chain transfer polymerization. Polym. Chem..

[81] Kushan E., Senses E. (2021). Thermoresponsive and Injectable Composite Hydrogels of Cellulose Nanocrystals and Pluronic F127. ACS Appl. Bio Mater..

[82] Pippa N., Psarommati F., Pispas S., Demetzos C. (2013). The shape/morphology balance: A study of stealth liposomes via fractal analysis and drug encapsulation. Pharm. Res..

[83] Perinelli D.R., Casettari L., Cespi M., Fini F., Man D.K.W., Giorgioni G., Canala S., Lam J.K.W., Bonacucina G., Palmieri G.F. (2016). Chemical–physical properties and cytotoxicity of N-decanoyl amino acid-based surfactants: Effect of polar heads. Colloids Surf. A Physicochem. Eng. Asp..

[84] Mulet X., Boyd B.J., Drummond C.J. (2013). Advances in drug delivery and medical imaging using colloidal lyotropic liquid crystalline dispersions. J. Colloid. Interface Sci..

[85] Pippa N., Naziris N., Stellas D., Massala C., Zouliati K., Pispas S., Demetzos C., Forys A., Marcinkowski A., Trzebicka B. (2019). PEO-b-PCL grafted niosomes: The cooperativilty of amphiphilic components and their properties in vitro and in vivo. Colloids Surf. B Biointerfaces.

[86] Zhou Z., Guo F., Wang N., Meng M., Li G. (2018). Dual pH-sensitive supramolecular micelles from star-shaped PDMAEMA based on β-cyclodextrin for drug release. Int. J. Biol. Macromol..

[87] Salatin S., Maleki Dizaj S., Yari Khosroushahi A. (2015). Effect of the surface modification, size, and shape on cellular uptake of nanoparticles. Cell Biol. Int..

[88] Avachat A.M., Bornare P.N., Dash R.R. (2011). Sustained release microspheres of ropinirole hydrochloride: Effect of process parameters. Acta Pharm..

[89] Keck T., Leiacker R., Riechelmann H., Rettinger G. (2000). Temperature profile in the nasal cavity. Laryngoscope.

[90] Hussein N., Omer H., Ismael A., Albed Alhnan M., Elhissi A., Ahmed W. (2020). Spray-dried alginate microparticles for potential intranasal delivery of ropinirole hydrochloride: Development, characterization and histopathological evaluation. Pharm. Dev. Technol..

[91] Marttin E., Verhoef J.C., Romeijn S.G., Merkus F.W. (1995). Effects of absorption enhancers on rat nasal epithelium in vivo: Release of marker compounds in the nasal cavity. Pharm. Res..

[92] Pailla S.R., Talluri S., Rangaraj N., Ramavath R., Challa V.S., Doijad N., Sampathi S. (2019). Intranasal Zotepine Nanosuspension: Intended for improved brain distribution in rats. DARU J. Pharm. Sci..

[93] Saha P., Kathuria H., Pandey M.M. (2023). Nose-to-brain delivery of rotigotine redispersible nanosuspension: In vitro and in vivo characterization. J. Drug Deliv. Sci. Technol..

[94] Chatzitaki A.T., Jesus S., Karavasili C., Andreadis D., Fatouros D.G., Borges O. (2020). Chitosan-coated PLGA nanoparticles for the nasal delivery of ropinirole hydrochloride: In vitro and ex vivo evaluation of efficacy and safety. Int. J. Pharm..

[95] Pardeshi C.V., Belgamwar V.S. (2017). Ropinirole-dextran sulfate nanoplex for nasal administration against Parkinson’s disease: In silico molecular modeling and in vitro-ex vivo evaluation. Artif. Cells Nanomed. Biotechnol..

[96] Jafarieh O., Md S., Ali M., Baboota S., Sahni J.K., Kumari B., Bhatnagar A., Ali J. (2015). Design, characterization, and evaluation of intranasal delivery of ropinirole-loaded mucoadhesive nanoparticles for brain targeting. Drug Dev. Ind. Pharm..

[97] Pardeshi C.V., Belgamwar V.S., Tekade A.R., Surana S.J. (2013). Novel surface modified polymer-lipid hybrid nanoparticles as intranasal carriers for ropinirole hydrochloride: In vitro, ex vivo and in vivo pharmacodynamic evaluation. J. Mater. Sci. Mater. Med..

[98] Wang Y., Zheng Y., Zhang L., Wang Q., Zhang D. (2013). Stability of nanosuspensions in drug delivery. J. Control. Release Off. J. Control. Release Soc..

[99] Mikušová V., Mikuš P. (2021). Advances in Chitosan-Based Nanoparticles for Drug Delivery. Int. J. Mol. Sci..

[100] de Souza Teixeira L., Vila Chagas T., Alonso A., Gonzalez-Alvarez I., Bermejo M., Polli J., Rezende K.R. (2020). Biomimetic Artificial Membrane Permeability Assay over Franz Cell Apparatus Using BCS Model Drugs. Pharmaceutics.

[101] Colombo G., Bortolotti F., Chiapponi V., Buttini F., Sonvico F., Invernizzi R., Quaglia F., Danesino C., Pagella F., Russo P. (2016). Nasal powders of thalidomide for local treatment of nose bleeding in persons affected by hereditary hemorrhagic telangiectasia. Int. J. Pharm..

