# Supplementary material for: Fabricating Polymer/Surfactant/Cyclodextrin Hybrid Particles for Possible Nose-to-Brain Delivery of Ropinirole Hydrochloride: In Vitro and Ex Vivo Evaluation
Source: Int J Mol Sci. 2024 Jan 18;25(2):1162. doi: 10.3390/ijms25021162 (PMC10816138; doi:10.3390/ijms25021162)
Supplement: Supplementary file 1 [file ijms-25-01162-s001.zip › ijms-2815107-supplementary.pdf]

SUPPLEMENTARY MATERIALS

Fabricating Polymer/Surfactant/Cyclodextrin Hybrid Particles for Possible Nose-To-Brain Delivery of Ropinirole Hydrochloride: In Vitro and Ex Vivo Evaluation

Elmina-Marina Saitani [1], Natassa Pippa [1], Diego Romano Perinelli [2], Aleksander Forys [3], Paraskevi Papakyriakopoulou [1], Nefeli Lagopati [4,5], Giulia Bonacucina [2], Barbara Trzebicka [3], Maria Gazouli [4], Stergios Pispas [6] and Georgia Valsami [1,*]

[1] Department of Pharmacy, School of Health Sciences, National and Kapodistrian University of Athens, Panepistimiopolis, 15771 Zografou, Greece; elminasait@pharm.uoa.gr (E.-M.S.); natpippa@pharm.uoa.gr (N.P.); ppapakyr@pharm.uoa.gr (P.P.); valsami@pharm.uoa.gr (G.V.)

[2] School of Pharmacy, Chemistry Interdisciplinary Project (CHIP), University of Camerino, Via Madonna delle Carceri, 62032 Camerino, Italy; diego.perinelli@unicam.it (D.R.P.); giulia.bonacucina@unicam.it (G.B.)

[3] Centre of Polymer and Carbon Materials, Polish Academy of Sciences, 34, M. Curie-Skłodowskiej St, 41-819 Zabrze, Poland; aforys@cmpw-pan.pl (A.F.); barbara.trzebicka@cmpw-pan.edu.pl (B.T.)

[4] Laboratory of Biology, Department of Basic Medical Science, School of Medicine, National and Kapodistrian University of Athens, 11527 Athens, Greece; nlagopati@med.uoa.gr (N.L.); mgazouli@med.uoa.gr (M.G.)

[5] Biomedical Research Foundation, Academy of Athens, 11527 Athens, Greece

[6] Theoretical and Physical Chemistry Institute, National Hellenic Research Foundation, 48 Vassileos Constantinou Avenue, 11635 Athens, Greece; pispas@eie.gr

* Correspondence: valsami@pharm.uoa.gr

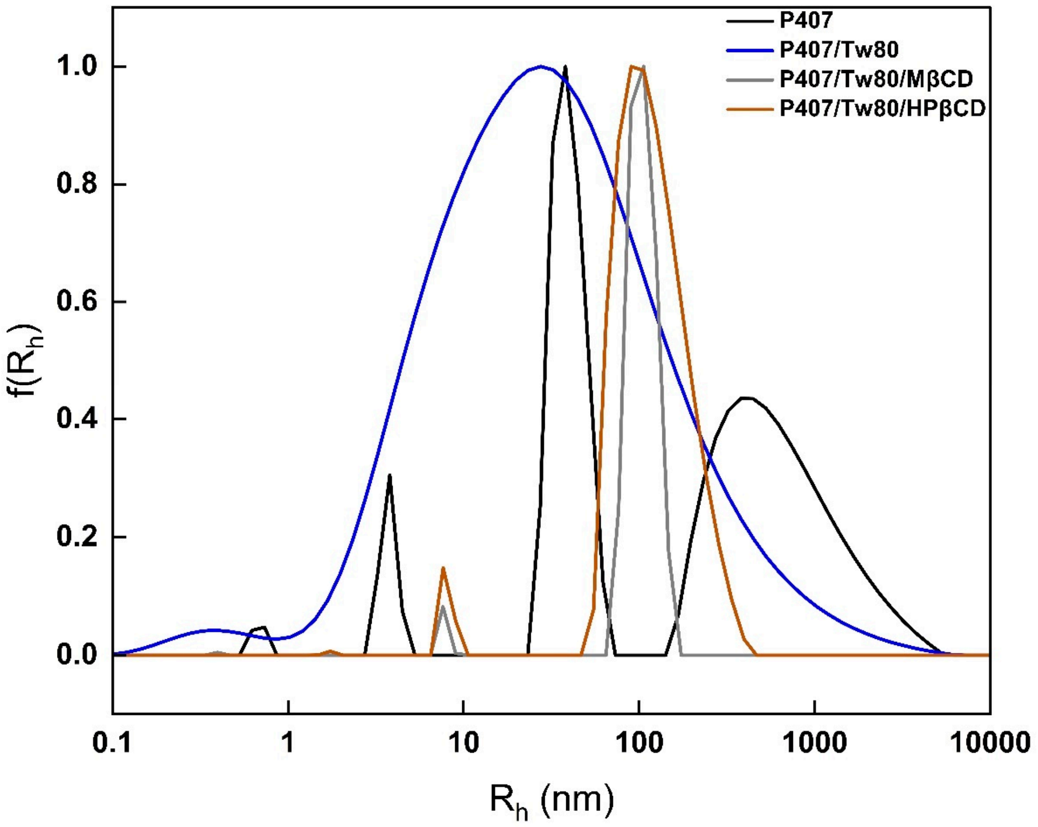

Figure S1. Size distributions for P407, P407/Tw80, P407/Tw80/MβCD, and P407/Tw80/HPβCD systems in aqueous dispersions prepared using the thin-film hydration method (t=0 days).

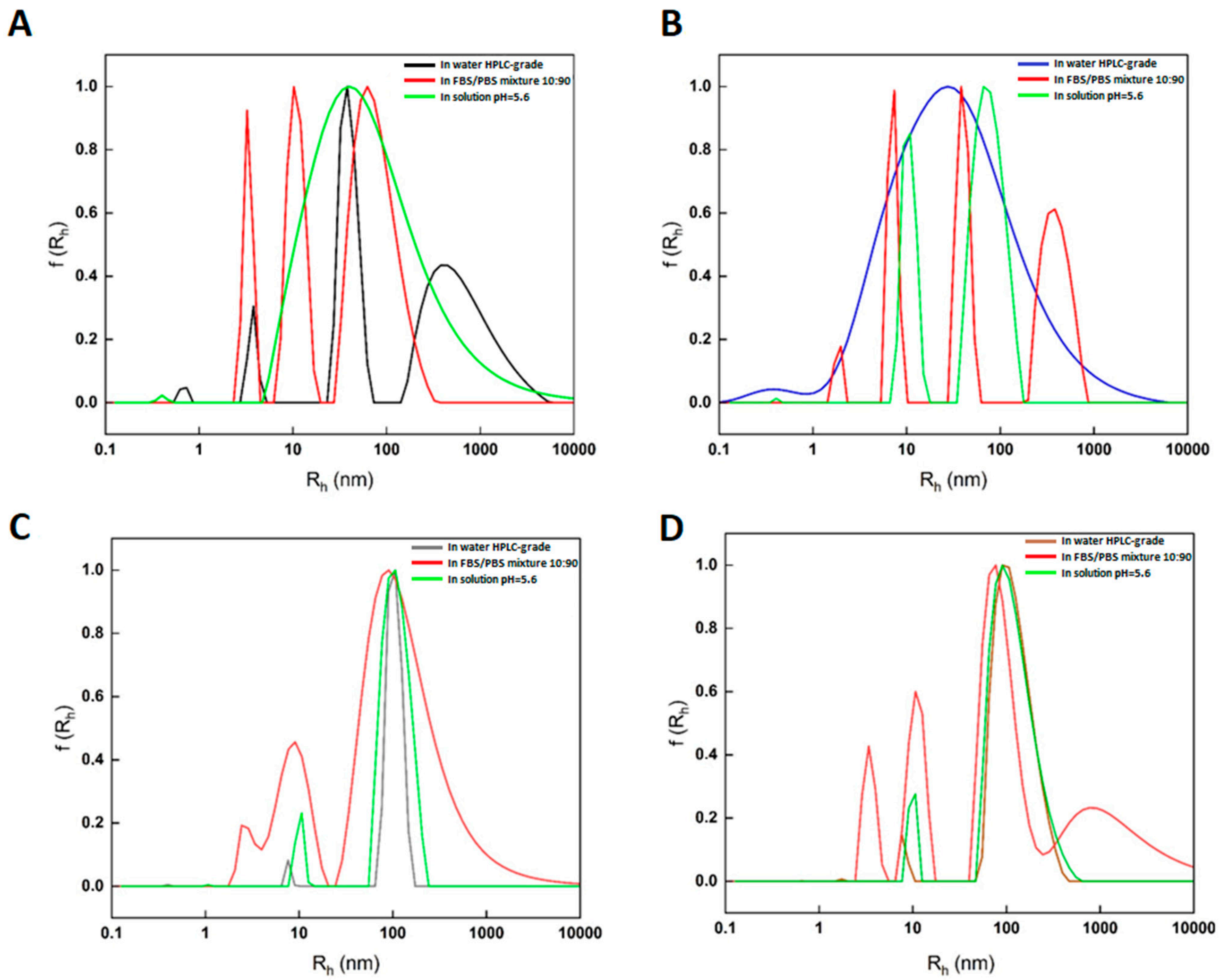

Figure S2. Size distributions for (**A**) P407, (**B**) P407/Tw80, (**C**) P407/Tw80/MβCD and (**D**) P407/Tw80/HPβCD systems in different dispersion media prepared using the thin-film hydration method (t=0 days).

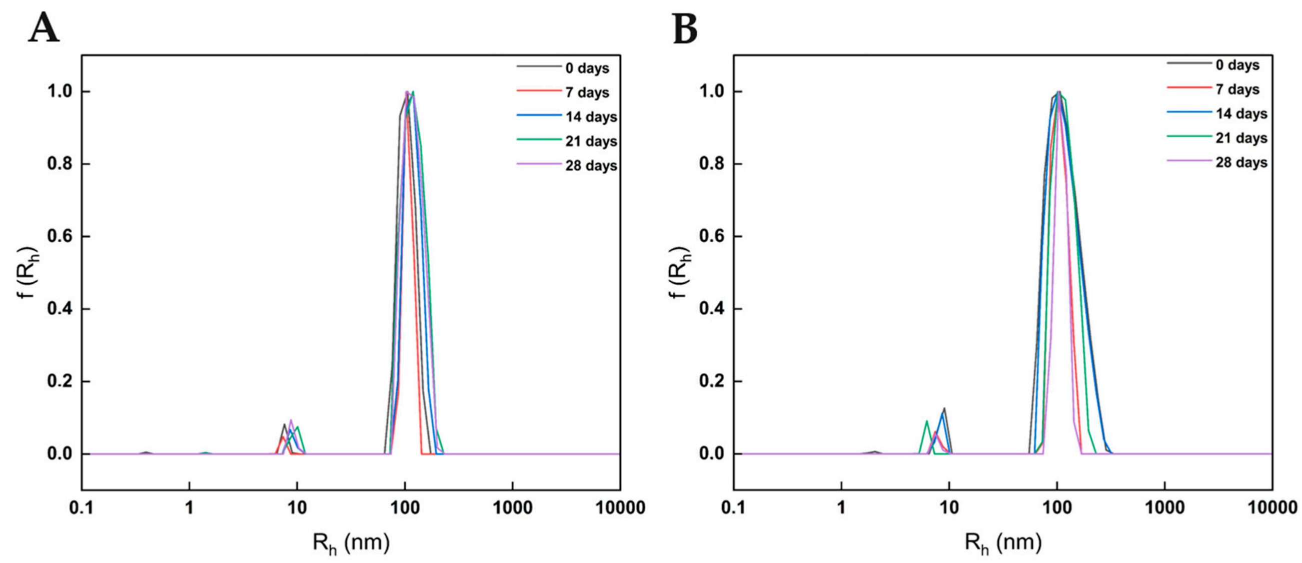

Figure S3. Stability assessment of (**A**) P407/Tw80/MβCD and (**B**) P407/Tw80/HPβCD hybrid systems.

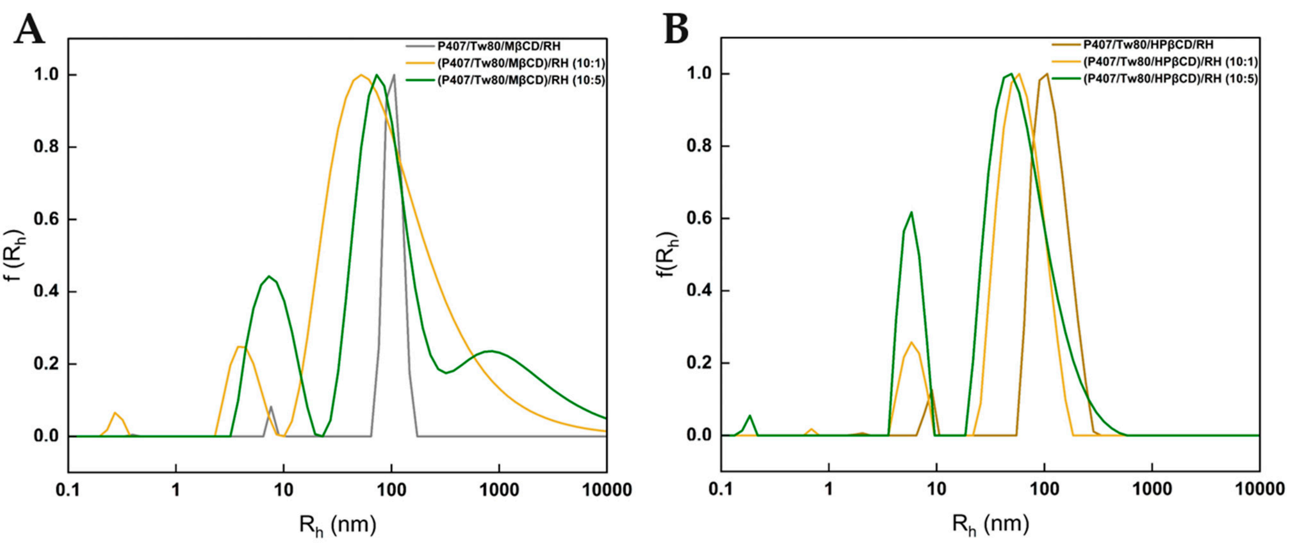

Figure S4. Size distribution for the (**A**) P407/Tw80/MβCD system, (P407/Tw80/MβCD)/RH in weight ratios of 10:1 and 10:5 and (**B**) P407/Tw80/HPβCD system, (P407/Tw80/HPβCD)/RH in weight ratios of 10:1 and 10:5 in aqueous dispersions prepared using the thin-film hydration method (t=0 days).

Table S1. Calorimetric heating profiles of pure compounds and their mixtures at the solid state. These calorimetric parameters correspond to the thermograms in Figure 2.

Sample	$T_{onset,m}$ (°C)[a]	T_m (°C)[b]	$\Delta T_{1/2,m}$ (°C)[c]	ΔH_m (KJ mol^{-1})[d]	$T_{onset,s}$ (°C)	T_s (°C)	$\Delta T_{1/2,s}$ (°C)	ΔH_s (KJ mol^{-1})	$T_{onset,t}$ (°C)	T_t (°C)	$\Delta T_{1/2,t}$ (°C)	ΔH_t (KJ mol^{-1})
P407	53.06	57.17	4.25	-46.38	151.56	157.83	6.53	7.83	–	–	–	–
Tw80	25.43	59.50	39.05	-2.07	–	–	–	–	–	–	–	–
MβCD	145.13	179.50	17.63	-1.29	68.20	75.33	6.91	-0.02	–	–	–	–
HPβCD	142.17	147.33	20.30	-5.70	–	–	–	–	–	–	–	–
Tw80/MβCD	123.78	147.50	27.10	0.50	184.83	187.00	6.65	-1.07	–	–	–	–
Tw80/HPβCD	121.29	132.33	16.19	-0.74	–	–	–	–	–	–	–	–
P407/Tw80	45.28	52.83	6.11	-31.08	–	–	–	–	–	–	–	–
P407/MβCD	46.19	52.83	5.01	-33.54	145.37	181.33	98.99	109.92	–	–	–	–
P407/HPβCD	47.63	52.83	4.22	-36.08	155.03	162.33	7.92	9.85	–	–	–	–
P407/Tw80/MβCD	46.14	53.00	7.24	-38.79	140.27	153.33	12.90	9.98	207.45	210.00	7.33	-3.42
P407/Tw80/HPβCD	50.20	55.17	5.92	-32.29	–	–	–	–	–	–	–	–

[a]T_{onset}: the temperature at which the thermal event starts.
[b]T: the temperature at which heat capacity (ΔC_P) at constant pressure is maximum.
[c]$\Delta T_{1/2}$: half width at the half peak height of the transition.
[d]ΔH: transition enthalpy normalized per mol of each system. m: main transition; s: secondary; t: ternary transition.

Table S2. The physicochemical characteristics of hybrid systems in A. FBS/PBS mixture (10:90) and B. buffer solution (pH=5.6 at the temperature of 34 °C).

A. Dispersed in FBS/PBS mixture

**Colloidal dispersions**	w/w	$R_{h\ (Cumulant)}$ (nm)[1]	PDI[2]	Number of peaks	$R_{h\ (Contin)}$ (nm)[3]	Weight of Peak (%)
P407	-	47	0.4_8	3	1) 3<br>2) 11<br>3) 75	1) 13%<br>2) 27%<br>3) 60%
P407/Tw80	70:30	38	0.5_0	1	1) 2<br>2) 7<br>3) 40<br>4) 389	1) 4%<br>2) 24%<br>3) 31%<br>4) 41%
(P407/Tw80)/MβCD	80:20	95	0.4_9	2	1) 8<br>2) 104	1) 3%<br>2) 97%
(P407/Tw80)/HPβCD	80:20	83	0.4_9	2	1) 9<br>2) 114	1) 3%<br>2) 97%

B. Dispersed in buffer solution pH=5.6 at 34 °C

**Colloidal dispersions**	w/w	$R_{h\ (Cumulant)}$ (nm)[1]	PDI[2]	Number of peaks	$R_{h\ (Contin)}$ (nm)[3]	Weight of Peak (%)
P407	-	42	0.5_0	3	62	100%
P407/Tw80	70:30	39	0.5_0	1	1) 11<br>2) 96	1) 27%<br>2) 73%
(P407/Tw80)/MβCD	80:20	90	0.4_1	2	1) 10<br>2) 108	1) 6%<br>2) 94%
(P407/Tw80)/HPβCD	80:20	86	0.4_2	2	1) 10<br>2) 118	1) 7%<br>2) 94%

[1] R_h indicates the average hydrodynamic radius of three replicates of each sample obtained by the Cumulant method

[2] PDI indicates the average polydispersity index, and the first decimal number is the significant one

[3] R_h indicates the average hydrodynamic radius of three replicates of each sample obtained by the Contin method

Table S3. Calorimetric parameters of pure RH, ternary systems and (P407/Tw80/CD)/RH at different weight ratios (10:0.1; 10:0.5; 10:1; 10:5; 10:10) at the solid state, using MβCD or HPβCD. These calorimetric parameters correspond to the thermograms of Figure 6.

Sample	Weight ratio	$T_{onset,m}$ (°C)[a]	T_m (°C)[b]	$\Delta T_{1/2,m}$ (°C)[c]	ΔH_m (KJmol^{-1})[d]	$T_{onset,s}$ (°C)	T_s (°C)	$\Delta T_{1/2,s}$ (°C)	ΔH_s (KJmol^{-1})	$T_{onset,t}$ (°C)	T_t (°C)	$\Delta T_{1/2,t}$ (°C)	ΔH_t (KJmol^{-1})
P407/Tw80/MβCD	-	46.14	53.00	7.24	-18.62	140.27	153.33	12.90	4.79	207.45	210.00	7.33	-1.64
P407/Tw80/HPβCD	-	50.20	55.17	5.92	-32.29	–	–	–	–	–	–	–	
RH	-	244.62	248.50	3.59	-22.69	-	-	-	-	-	-	-	-
(P407/Tw80/MβCD)/RH	10:0.1	42.51	49.50	4.94	-11.28	208.38	220.83	50.62	7.49	-	-	-	-
(P407/Tw80/MβCD)/RH	10:0.5	41.13	48.50	6.67	-11.41	204.05	205.33	1.97	-1.42	-	-	-	-
(P407/Tw80/MβCD)/RH	10:1	44.15	50.17	5.39	-11.38	237.33	234.42	2.28	-1.48	-	-	-	-
(P407/Tw80/MβCD)/RH	10:5	42.49	49.83	7.69	-8.48	234.33	237.33	3.52	-7.49	-	-	-	-
(P407/Tw80/MβCD)/RH	10:10	42.09	48.50	6.37	-6.98	224.21	230.83	5.74	-9.12	240.52	241.17	1.32	-3.17
(P407/Tw80/HPβCD)/RH	10:0.1	46.76	50.00	4.02	-13.07	123.28	141.33	18.15	1.84	166.33	169.50	10.36	-4.24
(P407/Tw80/HPβCD)/RH	10:0.5	43.56	51.83	8.75	-12.00	–	–	–	–	–	–	–	–
(P407/Tw80/HPβCD)/RH	10:1	40.89	49.50	7.27	-11.40	117.94	143.83	29.49	4.33	234.72	237.50	5.27	-0.82
(P407/Tw80/HPβCD)/RH	10:5	40.15	48.17	6.31	-8.37	120.98	144.67	17.18	2.22	206.48	217.33	9.73	-6.31
(P407/Tw80/HPβCD)/RH	10:10	41.44	49.00	6.01	-6.12	214.00	225.67	14.20	-10.49	–	–	–	–

[a]T_{onset}: the temperature at which the thermal event starts.
[b]T: the temperature at which heat capacity (ΔC_p) at constant pressure is maximum.
[c]$\Delta T_{1/2}$: half width at the half peak height of the transition.
[d]ΔH: transition enthalpy normalized per mol of each system. m: main transition; s: secondary; t: trinary transition.

Table S4. The flux across the cellulose membrane (J_{CM}) (mean ± SD, n = 3), the flux (J_{NM}) (mean ± SEM, n = 4) and the apparent permeability (P_{app}) across the nasal mucosa barrier of formulations F1-F4 and RH solution (0.5 mg/mL, PBS pH = 5.6). R-square of regression analysis of the amount of the drug permeated per unit area vs time, across the cellulose membrane and the nasal mucosa barrier are included in the table [$R^2_{(CM)}$ and $R^2_{(NM)}$, respectively].

**Formulation (F)**	**J_{CM} (μg/cm²/min) ± SD**	**$R^2_{(CM)}$**	**J_{NM} (μg/cm²/min) ± SEM**	**$R^2_{(NM)}$**	**P_{app} (cm/min)**
F1	$4.9\times10^{-4} \pm 5.1\times10^{-5}$	0.9492 ± 0.0053	$2.0\times10^{-4} \pm 1.0\times10^{-5}$	0.9645 ± 0.0017	0.40
F2	$5.9\times10^{-4} \pm 7.5\times10^{-5}$	0.9256 ± 0.0077	$2.0\times10^{-4} \pm 1.0\times10^{-5}$	0.9911 ± 0.0008	0.40
F3	$5.5\times10^{-4} \pm 5.2\times10^{-5}$	0.9572 ± 0.0054	$1.7\times10^{-4} \pm 1.0\times10^{-5}$	0.9825 ± 0.0010	0.35
F4	$5.9\times10^{-4} \pm 8.9\times10^{-5}$	0.8978 ± 0.0092	$1.9\times10^{-4} \pm 1.0\times10^{-5}$	0.9950 ± 0.0006	0.39
RH solution	$6.0\times10^{-4} \pm 8.2\times10^{-5}$	0.9157 ± 0.0085	$1.4\times10^{-4} \pm 1.0\times10^{-5}$	0.9769 ± 0.0009	0.28
